# Replicate geographic transects across a hybrid zone reveal parallelism and differences in the genetic architecture of reproductive isolation

**DOI:** 10.1093/evlett/qraf009

**Published:** 2025-05-07

**Authors:** Georgy Semenov, Haley Kenyon, Erik Funk, William Anderson, Michael McQuillan, Joan Spinelli, Austin Russell, Noel Martinez, Alex Van Huynh, Alana Alexander, Rena Schweizer, Ethan Linck, Zachary Cheviron, Matt Carling, Timothy Roth, Mark Robbins, Amber Rice, Scott Taylor

**Affiliations:** Department of Ecology and Evolutionary Biology, University of Colorado Boulder, Boulder, CO, United States; Department of Ecology and Evolutionary Biology, University of Colorado Boulder, Boulder, CO, United States; San Diego Zoo Wildlife Alliance, Escondido, CA, United States; Department of Ecology and Evolutionary Biology, University of Colorado Boulder, Boulder, CO, United States; Department of Genetics, University of Pennsylvania, Philadelphia, PA, United States; Department of Biological Sciences, Lehigh University, Bethlehem, PA, United States; Department of Biological Sciences, Lehigh University, Bethlehem, PA, United States; Department of Biological Sciences, Lehigh University, Bethlehem, PA, United States; College of Sciences, DeSales University, Center Valley, PA, United States; Department of Anatomy, School of Biomedical Sciences, University of Otago, Dunedin, New Zealand; Division of Biological Sciences, University of Montana Missoula, Missoula, MT, United States; Department of Ecology, Montana State University, Bozeman, MT, United States; Division of Biological Sciences, University of Montana Missoula, Missoula, MT, United States; Department of Zoology and Physiology, University of Wyoming, Laramie, WY, United States; Biological Foundations of Behavior Program, Franklin and Marshall College, Lancaster, PA, United States; University of Kansas Biodiversity Institute, Lawrence, KS, United States; Department of Biological Sciences, Lehigh University, Bethlehem, PA, United States; Department of Ecology and Evolutionary Biology, University of Colorado Boulder, Boulder, CO, United States

**Keywords:** Hybridization, reproductive isolation, introgression, hybrid zones, black-capped chickadee, Carolina chickadee

## Abstract

Determining the genetic architecture of traits involved in adaptation and speciation is one of the key components of understanding the evolutionary mechanisms behind biological diversification. Hybrid zones provide a unique opportunity to use genetic admixture to identify traits and loci contributing to partial reproductive barriers between taxa. Many studies have focused on the temporal dynamics of hybrid zones, but geographical variation in hybrid zones that span distinct ecological contexts has received less attention. We address this knowledge gap by analyzing hybridization and introgression between black-capped and Carolina chickadees in two geographically remote transects across their extensive hybrid zone, one located in eastern and one in central North America. Previous studies demonstrated that this hybrid zone is moving northward as a result of climate change but is staying consistently narrow due to selection against hybrids. In addition, the hybrid zone is moving ~5× slower in central North America compared to more eastern regions, reflecting continent-wide variation in the rate of climate change. We use whole genome sequencing of 259 individuals to assess whether variation in the rate of hybrid zone movement is reflected in patterns of hybridization and introgression, and which genes and genomic regions show consistently restricted introgression in distinct ecological contexts. Our results highlight substantial similarities between geographically remote transects and reveal large Z-linked chromosomal rearrangements that generate measurable differences in the degree of gene flow between transects. We further use simulations and analyses of climatic data to examine potential factors contributing to continental-scale nuances in selection pressures. We discuss our findings in the context of speciation mechanisms and the importance of sex chromosome inversions in chickadees and other species.

The evolution of reproductive barriers is key to the generation of new species (Coyne & Orr, 2002). Reproductive isolation between populations can arise through multiple mechanisms and in different geographic contexts but, in the majority of cases, a period of allopatry is essential (Coyne & Orr, 2002; Mayr, 1942; Price, 2008). During this period, populations accumulate genetic differences through drift and selection in the absence of gene flow, increasing the likelihood of speciation. However, complete geographic and genetic isolation is uncommon. In an increasing number of cases, it appears that gene flow occurred at some point during the divergence history of numerous species pairs and groups (Harrison, 1990; Harrison & Larson, 2014; Seehausen, 2004; Taylor & Larson, 2019), including humans (e.g., Dannermann et al., 2016; Dutheil et al., 2015; Harris et al., 2023; Sankararaman et al., 2014). Regardless of the mechanism by which reproductive isolation evolves, divergent lineages may come back into prolonged secondary contact where reproductive barriers are tested, with multiple potential outcomes (Abbot, 2013; Arnold, 2015; Barton, 2013; Payseur & Rieseberg, 2016).

Hybridization is one such outcome of secondary contact, leading to the formation of hybrid zones when divergent lineages exchange genes in a restricted area of sympatry (Abbot et al., 2016; Harrison & Larson, 2014; Peñalba et al., 2024). Ongoing hybridization allows for the investigation of reproductive barriers, leading to an expanded understanding of the factors that promote or erode them. The study of hybrid zones and hybridization can also uncover the mechanisms that underlie reduced fitness caused by hybrid genotypes and has allowed for the dissection of the genetic basis of adaptation and divergence (e.g., Jones et al., 2018; Poelstra et al., 2014; Semenov et al., 2021; Song et al., 2011; Toews et al., 2016; Turner & Harr, 2014). Importantly, hybrid zones are dynamic systems within which the extent of hybridization can vary across space and time.

Replicate temporal sampling across hybrid zones has revealed that hybrid zone characteristics (e.g., spatial position or frequency of hybridization) can change over time in response to environmental change (e.g., climate change or anthropogenic habitat transformation) (Aguillon & Rohwer, 2022; Ålund et al., 2023; Buggs, 2007; Dougherty & Carling, 2024; Grabenstein et al., 2023; Taylor, White, et al., 2014; Taylor et al., 2015; Ryan et al., 2018—but see Mettler & Spellman, 2009; Wang et al., 2019). Interestingly, while patterns of hybridization can similarly vary geographically in distinct parts of the same hybrid zone, the underlying causes of these differences have received less attention in the literature (but see Fraïsse et al., 2014; Larson et al., 2013; Mandeville et al., 2022; McFarlane et al., 2024; Natola et al., 2023; Teeter et al., 2010). Studying multiple geographically distinct transects across hybrid zones should help assess the extent to which hybridization is predictable or contingent on history and local conditions, helping pinpoint ecological factors that constrain or promote hybridization. In addition, replicate transects can illuminate which genes or genomic regions consistently contribute to reproductive isolation in different ecological contexts and hence play a primary role in the maintenance of species boundaries.

Black-capped (*Poecile atricapillus*) and Carolina chickadees (*Poecile carolinensis*) are sister species of passerine birds that hybridize in a spatially expansive zone of contact across eastern and central North America (Figure 1). The black-capped/Carolina chickadee hybrid zone is one of the best-studied examples of rapid (~1.2 km a year), climate-mediated northward zone movement: the contact zone east of the Appalachian Mountains closely tracks average winter temperatures, which are increasing as a result of climate change (Taylor, White, et al., 2014). Despite ongoing hybridization (Bronson, 2002; Bronson, Grubb, Braun, et al., 2003; Bronson, Grubb, Sattler, et al., 2003; Bronson et al., 2005; Davidson et al., 2013; Reudink et al., 2006, 2007; Taylor, Curry, et al., 2014; Taylor, White, et al., 2014), there is documented selection against individuals with admixed ancestry via reduced hatching success (Bronson, Grubb, Sattler et al., 2003, Bronson et al. 2005; Van Huynh & Rice, 2019), and probable developmental and physiological breakdown in hybrids (Driver et al., 2022; McQuillan et al., 2018; Wagner et al., 2020). Evidence for metabolic dysfunction comes from both experimental results (Olson et al., 2010) and patterns in genomic data (Taylor, Curry, et al., 2014; Wagner et al., 2020), which suggest that admixed individuals (hybrids and backcrosses) suffer from metabolic inefficiency. Such reduced metabolic efficiency likely increases the likelihood of overwinter mortality, as seen in other small passerines (Petit et al., 2017).

**Figure 1.**
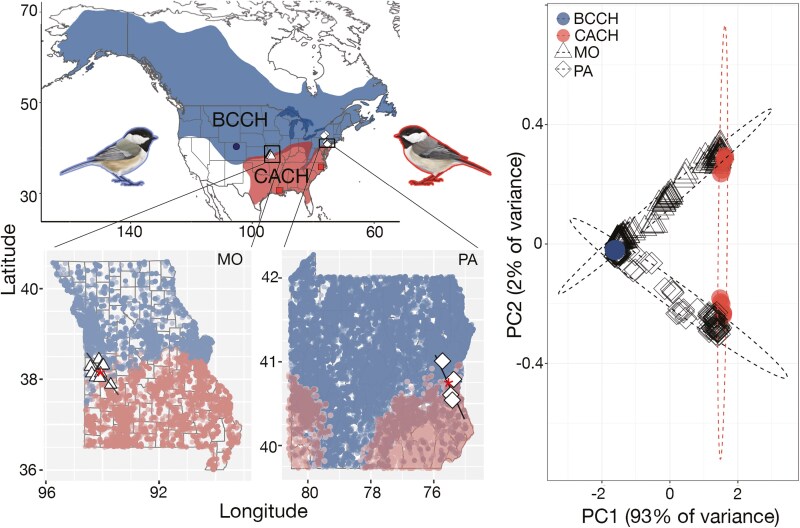
Geographic context of the study: Geographic distribution, sampling, and population structure as inferred by Principal Component Analysis in allopatric black-capped chickadees (*n* = 15, blue circles), Carolina chickadees (*n* = 11, red rectangles), and transects through their hybrid zones in Missouri (triangles, *n* = 131) and Pennsylvania (diamonds, *n* = 102). Distribution maps in inserts are based on eBird (release of August 2020) records. For geographic cline analysis, the position of sampling localities was projected on the perpendicular line across each region of the hybrid zone to estimate linear distances. Asterisks show the inferred center of each geographic cline. Note that there are samples of black-capped chickadees collected directly north from the hybrid zone (New York State, Supplementary Table S1) that were used as “allopatric” in the analysis of the putative Z chromosome inversion that compares western and eastern black-capped chickadee populations.

Another intriguing mechanism of selection against hybrid chickadees involves cognitive breakdown. During the winter months, particularly in harsher climates, several chickadee species—including black-capped, and to a lesser extent, Carolina chickadees—rely heavily on cached food sources scattered throughout their home range (Smith, 1995; Sonnenberg et al., 2019). Recovering scatter-cached food requires exceptional spatial memory (Branch et al., 2022; Pravosudov et al., 2025), but black-capped and Carolina chickadee hybrids appear to experience deficiencies in learning and memory (McQuillan et al., 2018). Hybrids with reduced spatial memory and learning abilities likely have reduced fitness in the wild, particularly during cold winters, such that environmental selection on hippocampal function might contribute to partial reproductive isolation between black-capped and Carolina chickadees (McQuillan et al., 2018; Rice, 2020; Rice & McQuillan, 2018). This idea is supported by evidence that neuron-related gene ontology categories (e.g., glutamate signaling and synaptic transmission) were overrepresented in genomic regions that were highly differentiated and resistant to introgression between black-capped and Carolina chickadees across a 10-year period (Wagner et al., 2020).

While the eastern region of the expansive black-capped/Carolina chickadee hybrid zone (Figure 1) has been extensively studied, its central and western reaches have received less attention. Recent work by Alexander et al. (2022) showed that the rate of hybrid zone movement varies across longitude: in the state of Missouri (central USA, Robbins et al., 1986), the hybrid zone is moving five or six times slower (~0.2 km a year) than in the state of Pennsylvania. Of the potential factors that might contribute to geographic differences in the rate of hybrid zone movement, the velocity of climate change may be the most important (Alexander et al., 2022; McQuillan & Rice, 2015). In general, climate change is occurring more slowly in interior (continental) areas of North America than its coasts, a pattern that holds true when comparing Missouri to Pennsylvania (Alexander et al., 2022; McQuillan & Rice, 2015). It remains unclear, however, whether hybridization frequency and reproductive isolation are consistent over the large geographic scale of the chickadee hybrid zone. There is some evidence that dynamics vary regionally: previous work in Missouri did not find loci with restricted introgression that overlapped between replicate transects of the hybrid zone in Missouri and Pennsylvania (Alexander et al., 2022). However, Alexander et al. (2022) used a reduced-representation sequencing approach that covered only a small fraction of the genome. Furthermore, the authors did not explicitly compare the representation of distinct hybrid ancestry classes (F1s, advanced generation hybrids, and backcrosses) to assess if variation in the rate of hybrid zone movement translates into distinct patterns of hybridization. As such, the genetic basis of the physiological mechanisms mediating black-capped and Carolina chickadee hybridization range-wide remains unknown.

In this study, we use whole genome sequencing of 259 individuals to test for variation in hybridization dynamics in two geographically remote transects across the chickadee hybrid zone: one sampled in Pennsylvania (eastern USA) and the other in Missouri (central USA) (Figure 1). Considering that previous studies found differences in the rate of the hybrid zone movement across North America (Alexander et al., 2022) and implicated geographical variation in climate as the potential mechanism (McQuillan & Rice, 2015), we hypothesize that hybridization and introgression patterns should reflect these differences. Eastern parts of the hybrid zone should experience stronger selective pressures from climatic variables as they relate to factors affecting overwinter chickadee survival. We first ask whether patterns of hybridization (such as the width of the hybrid zone and the relative proportion of distinct hybrid classes) differ between the two transects. Second, we assess patterns of introgression to pinpoint genomic regions and loci with consistently restricted introgression. Third, we identify genes and biological processes that might contribute to reproductive barriers in both transects and explore the genome-wide architecture of differentiation and reproductive isolation between black-capped and Carolina chickadees. We conclude by using forward-time simulations and analyses of ecological variables to assess potential mechanisms behind the spatiotemporal dynamics of the hybrid zone.

## Methods

### Sampling

We sampled chickadees in two transects spanning portions of the hybrid zone in Missouri (*n* = 131, same samples used in Alexander et al. (2022) from 2016 to 2019) and Pennsylvania (*n* = 102), as well as distant allopatric populations of black-capped (*n* = 15) and Carolina (*n* = 11) chickadees from Colorado and Louisiana/North Carolina, respectively (Figure 1, Supplementary Table S1). In addition to facilitating our comparison of hybrid zone dynamics, sampling multiple populations allowed us to account for intrapopulation genetic variation in the Carolina chickadee (i.e., distinct mtDNA lineages in the central and eastern parts of North America) (Gill et al., 1993; Supplementary Table S1). Chickadees were trapped in the field using mist nets.

### Whole genome sequencing and characterizing patterns of divergence and hybridization

We sequenced whole genomes, aiming for 15× read depth and prepared genotype tables following commonly used protocols (see Supplementary Methods for details). We assessed inter- and intraspecific genetic structure with Principal Component Analysis (PCA). We performed PCA on all species, populations, and hybrids using the R v.3.6.1 function *prcomp()* (Rstudio v.1.1.453) and a mean-centered genetic covariance matrix of SNP genotypes generated from a VCF file that was thinned to reduce the likelihood of physical linkage (1 variant site per 10 kb window). Next, we used gghybrid v. 2.0.0 (Bailey, 2023) to estimate individual hybrid indexes. To do so, we first calculated per-SNP Weir and Cockerham’s *F*_ST_ between allopatric black-capped (*n* = 15) and Carolina (*n* = 11) chickadees using *vcftools* (Danecek et al., 2011). We then selected SNPs with alleles fixed to alternative states (*F*_ST_ = 1), further thinned this dataset to retain 1 SNP per 10 kb window (9,634 loci total), and input the dataset to gghybrid’s *esth()* function. We discarded the first 5,000 iterations as burn-in, using the subsequent 10,000 iterations to calculate the posterior probability of hybrid index values.

We also used gghybrid to run a Bayesian Genomic Cline (BGC) analysis. This analysis allowed us to quantify transitions in allele frequencies of individual SNPs compared to genome-wide background ancestry and identify loci with restricted introgression. We used the function *ggcline()* on a dataset including all loci with *F*_ST_ = 1 (*n* = 296,996) and identical MCMC parameters. In downstream analyses, we focused on loci with a cline steepness (*v*) parameter exceeding the value of 1 and *p* < 0.05. In *gghybrid*, *v* = 1 indicates that a given locus has a rate of transition not significantly different from the genome-wide average, while *v* > 1 indicates a cline with a logistic (or sigmoidal) form. This shape indicates restricted gene flow on either side of the cline center (Bailey, 2023), analogous to the *R* parameter in Gompert and Buerkle (2012). Higher *v* values indicate comparatively more restricted introgression (Bailey, 2023). We then used the annotation from Semenov et al. (2024) to match introgression-resistant loci to gene models, including regions up to 5,000 bp before and after reading frames to account for potential regulatory substitutions.

Previous studies revealed that black-capped and Carolina chickadees belong to divergent mtDNA clades, with additional mtDNA segregation between western and eastern Carolina chickadees (Gill et al., 1993). To assess the transition in mtDNA haplotypes, we aligned sequenced reads to the complete mitochondrial genome of the black-capped chickadee (Wagner et al., 2020) and implemented PCA as described above to identify mtDNA clusters and to assign individual mitotypes based on their grouping with allopatric samples.

To characterize the width of the hybrid zone across each transect, we used the geographic cline analysis implemented in *hzar* v.0.2-5 R package (Derryberry et al., 2014). We used PC1 scores from the PCA (as described above) as a proxy for genomic ancestry and to fit empirical data corresponding to five geographic cline models using the Metropolis–Hastings MCMC algorithm as described in Semenov et al. (2021). To characterize the distance along each transect, the geographic location of sampling coordinates (Supplementary Table S1) was projected on a line approximating a 90-degree transect across the hybrid zone as shown in Figure 1. To characterize genome-wide patterns of differentiation, we estimated *F*_ST_ in 25 kb sliding windows with a 5 kb step in three comparisons: allopatric samples, black-capped vs. Carolina chickadee in Missouri (West), and black-capped vs. Carolina chickadee in Pennsylvania (East). For the latter two comparisons, we selected the least admixed individuals with hybrid indices of <0.1 or >0.9 and heterozygosity below 0.1.

### Detection of Z chromosome inversions

Patterns of *F*_ST_ between chickadee populations suggested potential chromosomal inversions (see *Result*s) both between black-capped and Carolina chickadees (Inversion 1) and between western and eastern Carolina chickadee populations (Inversion 2). To further characterize these regions, we used *asaph* v.2.0 (Nowling et al., 2022) to generate local PCAs and test associations between SNPs and PCA coordinates. *Asaph* identifies SNPs highly associated with PCA coordinates and then visualizes these scores as a Manhattan plot. Higher values indicate stronger associations between SNP genotype and PCA location. Inversions generally appear as stepwise patterns of SNP-PCA associations (i.e., do not show gradual transitions along the *x*-axis).

To focus our inversion exploration, we first subset our data to include only the Z chromosome (scaffold Z_1, representing over 90% of the total chromosome length). This comparison included western allopatric black-capped chickadee (*n* = 23 from Colorado) and western Carolina chickadee (*n* = 5) from Louisiana. Further, we included eastern allopatric black-capped chickadee populations (*n* = 13 from New York State, a sampling site adjacent to the northern edge of the hybrid zone, see Figure 1 and Supplementary Table S1). Note that this reference population was used as allopatric only in the analysis of putative Z chromosome inversions. Finally, we analyzed six Carolina chickadees from eastern populations (South Carolina). We then characterized different inversion haplotypes within the two transects with PCAs. To do so, we first subset the data to independently examine Inversion 1 haplotypes within each transect (eastern transect *n* = 106, western transect *n* = 136), removing sites on the Z chromosome that fell outside of the Inversion 1 boundaries (as estimated by *asaph*) and calculating inbreeding coefficients (*F*_IS_) for each individual within each transect to identify putative heterozygotes. We similarly subset our data to characterize the frequency and distribution of Inversion 2, retaining only sites within its boundaries and individuals with both copies of the Carolina chickadee Z chromosome. By removing all heterozygotes and black-capped chickadee individuals, the latter step allowed us to avoid clustering due to black-capped chickadee ancestry. Finally, we performed PCA as described above, expecting that if there was a completely segregating inversion between populations of Carolina chickadee, there would be full segregation of individuals by transect location.

### Ingenuity pathway analysis

To characterize the organization of genes with evidence of restricted introgression into developmental processes and regulatory pathways, we used the Ingenuity Pathway Analysis (IPA) software developed by QIAGEN (Release 2024-07-22, QIAGEN Inc., https://digitalinsights.qiagen.com/IPA (Krämer et al., 2014). Unlike methods that use unstructured gene lists for assessing overrepresentation via gene ontology categories, pathway analysis tools (such as IPA) can establish causal hierarchical relationships from gene lists and provide insights into biological mechanism, not just pattern (Semenov et al., 2024). The IPA database in particular combines insights from over 100,000 published datasets and >7,000,000 findings (gene associations or functions) to evaluate patterns of gene co-expression, hierarchical gene expression relationships, and connections between individual genes and a phenotype of interest. To assess overrepresentation of genes in higher-level pathways, we performed an Ingenuity Knowledge Base Core analysis with default settings (*p* < 0.05). We searched the complete list of unique genes with restricted introgression that overlapped between Pennsylvania and Missouri to assess (1) their connection to known phenotypes of interest and (2) their overrepresentation in the list of IPA Core biological pathways.

### Simulations of the hybrid zone dynamics and analysis of climate variation

Previous studies documented significant variation in the rate of hybrid zone movement in Missouri (slow; Alexander et al., 2022) compared to Pennsylvania (fast; Taylor, White, et al., 2014). These differential rates of movement are likely associated with differences in climate between the locations that may be relevant for selection against hybrids (i.e., potentially stronger selection in Pennsylvania due to harsher winter conditions). To explore both the influence of hybrid zone movement and selection on hybrid zone dynamics and to more thoroughly quantify differences in winter climate between the sampling transects, we performed additional analyses. First, we used forward-time simulations to (1) explore if the rate of hybrid zone movement itself can lead to variation in introgression patterns (i.e., alter the steepness of genomic clines) and (2) examine how varying levels of selection interact with hybrid zone movement. Second, we compared a series of climatic variables that may be relevant selective agents between Missouri and Pennsylvania. See Supplementary Methods for details.

## Results

### Similar patterns of hybridization between transects

**Figure 2.**
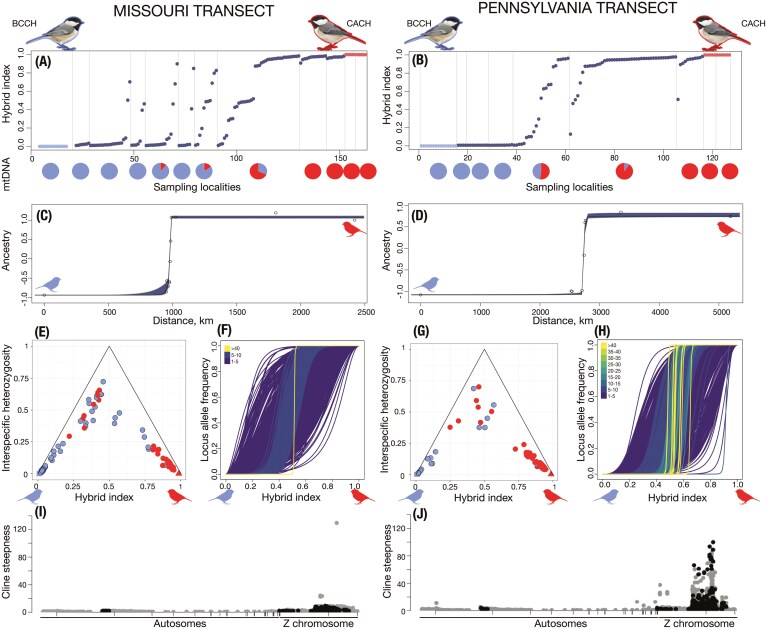
Genomic variation and patterns of introgression across two geographically distant transects in Missouri and Pennsylvania: (A) Hybrid index along the Missouri transect. (B) Hybrid index along the Pennsylvania transect. For both plots, the *x*-axis shows the cumulative number of individuals and the frequency of mtDNA haplotypes in each location is visualized as pie charts. (C) The best supported geographic cline with 95% CI for the Missouri transect. (D) The best supported geographic cline with 95% CI for the Pennsylvania transect. (E) Hybrid index vs. heterozygosity (red and blue indicate mtDNA types) in the Missouri transect. (F) Significant genomic clines in Missouri identified by BGC (colors indicate cline steepness values). (G) Hybrid index vs. heterozygosity (red and blue indicate mtDNA types) in the Pennsylvania transect. (H) Significant genomic clines in Pennsylvania identified by BGC (colors indicate cline steepness values). (I) The genomic distribution of all loci (*n* = 296,996) tested in BGC analysis for the Missouri transect. (J) The genomic distribution of all loci (*n* = 296,996) tested in BGC analysis for the Pennsylvania transect. For both plots, black shows SNPs with significantly reduced introgression in both Pennsylvania and Missouri. Light blue and red colors correspond to black-capped and Carolina chickadee ancestry states, respectively, throughout the figure.

After filtering, our whole genome dataset included 10,998,284 nuclear SNPs. We found strong signals of population structure, with the first axis of the PCA separating black-capped and Carolina chickadees and PC2 separating western and eastern Carolina chickadee populations (Figure 1). Similarly, consistent mtDNA clusters corresponding to the eastern and western Carolina chickadee populations and black-capped chickadee were recovered for variants (*n* = 212) extracted from the separate alignment to the black-capped chickadee mitochondrial reference genome (Supplementary Figure S1). There was a broad range of intermediate PC1 and PC2 values in the nuclear PCA corresponding to individuals with a continuum of admixed ancestry in both hybrid zones (Figure 1). All individuals from the reference (allopatric) parental samples of black-capped and Carolina chickadees were assigned to hybrid index values close to 0 or 1 (Figure 2A and B). Consistent with PCA results, hybrid index values ranged broadly, with few pure parental types and various intermediates in Missouri and Pennsylvania. These data support a scenario of ongoing hybridization and introgression in both transects (Figure 2A and B). Comparison of hybrid index versus heterozygosity (Figure 2E and G) illustrated that most admixed genotypes are the product of many generations of hybridization and backcrossing, with no individuals falling into the F1 category (individuals with heterozygosity of 1 and hybrid index of 0.5). While a fraction of individuals in both transects had hybrid indexes close to those found in allopatric populations, none had heterozygosity of 0, indicating some traces of admixture.

### Variation in hybrid zone width between transects

In Missouri, geographic cline analysis in *hzar* suggested asymmetrical introgression toward the black-capped chickadee (Figure 2C), supporting a model with a left-sided introgression tail. In Pennsylvania (Figure 2D), the best supported model was for symmetrical (or mirrored) introgression. The genome-wide cline estimated from the Missouri transect was significantly narrower (2.7 km, limits 0.27–4.7 km) compared to Pennsylvania (29.9 km, limits 23.4–33.6 km). However, there were secondary peaks in the Missouri cline width probability distribution around 30–40 km (Supplementary Figure S2), suggesting uncertainty. The transition of mtDNA haplotype frequencies closely coincided with that of nuclear loci across both transects (Figure 2A and B), suggesting concordant selection across nuclear and mitochondrial genomes. Interestingly, mtDNA haplotypes of both black-capped chickadees and Carolina chickadees were found among individuals with nuclear hybrid ancestry (Figure 2E and G); however, the vast majority of advanced generation backcrosses had mtDNA haplotypes that matched their majority nuclear background.

### Consistent signatures of restricted introgression between transects

**Figure 3.**
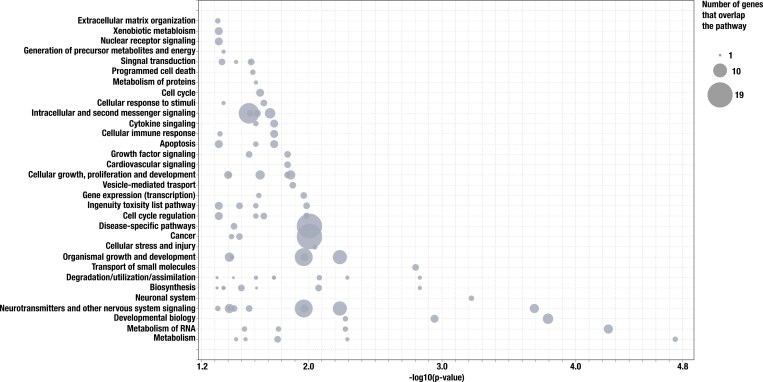
Significantly overrepresented biological pathways (IPA core database) for the subset of unique genes (*n* = 322) with restricted introgression in both Pennsylvania and Missouri transects.

Out of 296,996 SNPs fixed to alternative allelic states between allopatric black-capped and Carolina chickadees, 34,140 and 73,238 SNPs had clines significantly steeper than the genomic background in the Pennsylvania and Missouri transects (respectively). Of these loci, nearly all significant clines were clustered on the Z chromosome (*n* = 33,144, 97.0 %) in Pennsylvania and (*n* = 73,176, 99.9%) in Missouri (Figure 2F and H). This pattern could be driven by several alternative mechanisms such as strong linked selection on shared genomic architecture, suppressed recombination due to the physical properties of sex chromosomes, or large chromosomal rearrangements (see below). Again, largely driven by SNPs on the Z chromosome, cline steepness differed between the two transects (Figure 2I and J), with patterns in Missouri consistent with introgression being less restricted on average (mean steepness of 3.13) compared to Pennsylvania (mean steepness of 3.88). While mean steepness between the two transects varied by only ~20%, there are a large number of outlier loci with much greater steepness values in Pennsylvania (Figure 2F, H–J): In Missouri, there was only 1 SNP with a cline where *v* > 10, compared to 407 in Pennsylvania (Figure 2). Despite these differences, there was prominent overlap between the loci identified as significant outliers (*n* = 18,138) accounting for ~53% and ~25% of all outliers in Pennsylvania and Missouri, respectively. The breadth of overlap was even higher in steep clines from SNPs located around gene models. Out of 683 genes with restricted introgression in the Pennsylvania transect and 608 genes in the Missouri transect, 337 genes overlapped and 322 were unique. Genomic regions that were resistant to introgression in both transects were significantly overrepresented for genes with neurological and metabolic functions (Figure 3, Supplementary Figures S3 and S4). Of particular note, these genes were involved in the formation of the hippocampus, axonogenesis, regulation of basal metabolic rate, thermogenesis, regulation of hypothermia, and oxidative phosphorylation (Supplementary Figures S3 and S4), all of which are relevant with respect to previously identified or hypothesized mechanisms of hybrid breakdown (but see the caveat above).

### Variable divergence and multiple inversions on the Z chromosome

**Figure 4.**
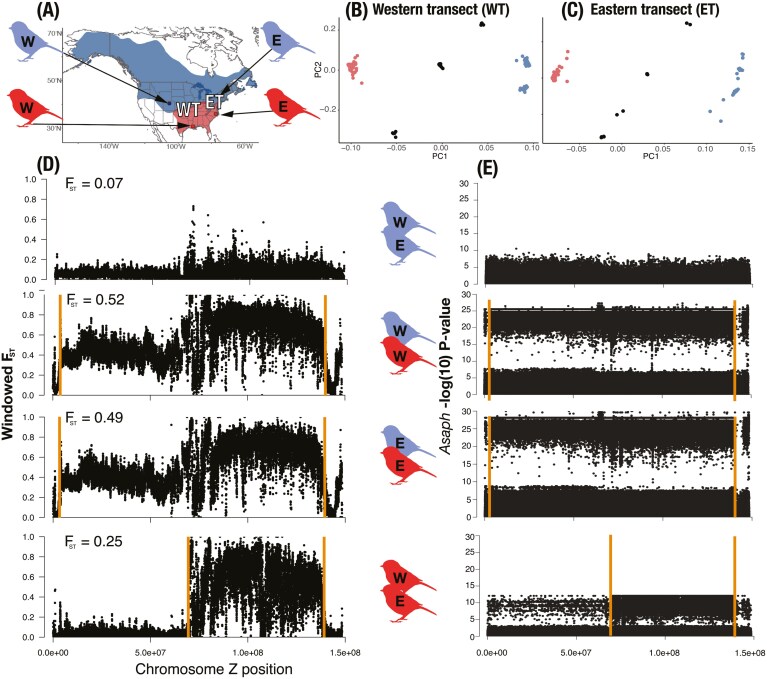
Differentiation on the Z chromosome and detection of the putative inversion. (A) Sampling locations of allopatric populations of western and eastern black-capped (blue) and Carolina (red) chickadees. WT and ET stand for western (Missouri) and eastern (Pennsylvania) transects. (B) and (C) PCA from the Z chromosome compared across the WT (89,123 SNPs) and ET (90,592 SNPs). Red, black, and blue colors show inversion states corresponding to Carolina chickadee, heterozygote, or black-capped chickadee respectively. (D) *F*_ST_ estimated in 25 kb sliding windows with a 5 kb step for the four allopatric comparisons, not including any transect samples. (E) Results of an *asaph* analysis to detect the chromosomal inversions between the same allopatric populations as in (D). Higher *y* axis values indicate a higher probability of being an inverted region. Orange lines in (D) and (E) show approximate break points of the inversion between black-capped and Carolina chickadees, as well as the two populations of Carolina chickadees.

Because the Z chromosome showed the most prominent differentiation and contained the majority of outliers, we further examined variation between and within species for the largest Z scaffold (Z_1), which encompassed over 90% of its total length (Figure 4). Populations of black-capped chickadees from Colorado and New York showed only low differentiation along the entire length of Z (Figure 4D, panel 1). In contrast, western and eastern populations of Carolina chickadees showed a distinct pattern of contrasting low and high differentiation in blocks that each spanned roughly half the chromosome (Figure 4D, panel 4). Comparisons between western black-capped and western Carolina chickadees and eastern black-capped and eastern Carolina chickadee populations showed generally high differentiation along the majority of Z chromosome, with a local maximum in the same region where differentiation peaked in comparisons of eastern and western Carolina chickadees (Figure 4D, panels 2 and 3).

*Asaph* identified two significant inversions between populations of chickadees. The first—a putative inversion (Inversion 1) between black-capped and Carolina chickadees—spanned most of the Z chromosome (Figure 4). This inversion was found independently in both transects and is 135.6 Mb long in the East (4,019,600 bp–139,615,224 bp) and 133.8 Mb long in the West (4,019,600 bp–137,849,599 bp). Importantly, *asaph* identified the left boundary of Inversion 1 in the same location in both independent transects. The 1.8 Mb difference in inversion length may be explained by either local recombination or potential short read data alignment errors. The second putative inversion identified by *asaph* (Inversion 2) was between the western and eastern populations of Carolina chickadee, spanning 68.5 Mb (70,257,022 bp–138,784,640 bp). Local PCA did not identify an inversion between allopatric populations of black-capped chickadees from Colorado and New York State (Figure 4). Most loci with significantly restricted introgression were found within the boundaries of Inversion 2: 93% in Missouri and 77% in Pennsylvania.

PCAs identified three inversion haplotype groups in each transect, which separated along the PC1 axis (Figure 4B and C, Supplementary Figure S5). The eastern transect included 32 individuals with both copies of the black-capped haplotype, 12 heterozygotes, and 62 individuals with both copies of the Carolina haplotype. The western transect included 57 individuals with both copies of the black-capped haplotype, 28 heterozygotes, and 51 individuals with both copies of the Carolina haplotype (Figure 4). *F*_IS_ values for the 28 individuals found in an intermediate position in the PCA were the lowest observed within the eastern transect. The same pattern was found in the western transect, where the 12 individuals in an intermediate position on PC1 also had the lowest *F*_IS_ scores. We found no overlap in inversion haplotype distributions, with all heterozygous or homozygous Carolina individuals in the eastern transect having the eastern inversion haplotype, and the western transect individuals all having the western haplotype. Additional variation along the PC2 axis in both transects was driven by females, suggesting some amount of recombination between inversion types of the noninverted (left arm) of the Z among Carolina chickadees (Figure 4B and C).

### Selection strength and hybrid zone movement both contribute to cline steepness

Our forward-time simulation revealed that while the rate of hybrid zone movement itself can contribute to the distinct patterns of introgression, selection strength plays a comparatively stronger role (see Supplementary Results for additional details).

### Environmental variables differ significantly between Pennsylvania and Missouri

We found significant differences in winter harshness climate variables that are likely relevant for selection against admixed chickadees. In brief, the locations around the hybrid zone center in Pennsylvania were colder, experienced more precipitation, and showed smaller daily temperature fluctuations compared to Missouri (see Supplementary Results for additional details).

## Discussion

We analyzed hybridization, introgression, and the factors that might influence their dynamics in two geographically distant transects across the hybrid zone between black-capped and Carolina chickadees. This hybrid zone is a particularly well-known example of a natural system influenced by climate change, which drives its rapid (~1.2 km per year, Taylor, Curry, et al., 2014) northward movement in Pennsylvania and slower (~0.2 km per year) northward movement in Missouri (Alexander et al., 2022). Yet to date, the mechanisms underlying differential rates of hybrid zone movement—and the potential effects of variation in hybrid zone movement on hybridization dynamics—have remained poorly understood.

Our analysis of genome-wide hybrid indexes revealed that hybridization patterns are broadly similar between Missouri and Pennsylvania. We detected no F1s and few parental genotypes in both transects, where the majority of individuals were advanced generation hybrids and backcrosses. Although the cline in genome-wide ancestry in Missouri was the narrower of the two transects, our estimate of its width included substantial uncertainty, with a secondary peak in the posterior probability distribution around 30 km—a figure similar to our cline width estimate for Pennsylvania. We further found that transitions in mitochondrial haplotype frequencies approximately coincided with the spatial transitions in nuclear markers across both transects and observed a near-perfect match between nuclear and mitochondrial types in advanced generation backcrosses. The set of SNPs that were resistant to introgression overlapped prominently between the two transects, and those located in genic regions were associated with previously known phenotypic traits likely involved in reduced hybrid fitness, including metabolic and neurological functions (Supplementary Figures S3 and S4).

Despite the similarities between the two transects, we found noticeable differences in per-locus (and more broadly, genomic region-specific) introgression rates on the Z-chromosome, with more restricted introgression in Pennsylvania. Patterns of differentiation on the Z revealed that the chromosome varies significantly between and within species. This pattern is particularly striking between western and eastern populations of Carolina chickadees, which may indicate the presence of chromosomal inversions. Indeed, we found support for two possible inversions: a larger inversion encompassing most of the Z chromosome and segregating between black-capped and Carolina chickadees, and a smaller inversion between western and eastern Carolina chickadee populations in the right arm of the Z chromosome. We found that distinct Carolina chickadee Z-inversion types are found in the Missouri and Pennsylvania transects, implicating their role in observed Z-specific introgression pattern variation between our two sampled transects. While there were a few heterozygous individuals for the inversion in both transects (Figure 4, Supplementary Figure S5), the modular nature of genomic variation in this region suggests little to no gene flow along the Z, as expected given the recombination suppressing effects of large chromosomal rearrangements. We further note that unlike Carolina chickadees, the studied populations of black-capped chickadees (from Colorado and New York) appear genetically very similar with mean whole genome *F*_ST_ = 0.052 and no *F*_ST_ windows with fixed differences (25 kb sliding windows with 5 kb step), including on the Z chromosome. We nonetheless emphasize that our choice of the Colorado sample as a reference population for the genomic cline analysis in both transects could have an effect (most likely minor) on cline steepness estimates.

Similar patterns of hybridization in geographically distinct parts of the hybrid zone suggest that broadly concordant selective pressures contribute to reproductive isolation between black-capped and Carolina chickadees range-wide. In both transects, the near-complete absence of F1s and pure parental genotypes indicates that hybridization has led to substantial admixture, with subsequent gametic segregation, recombination, and selection against hybrids and backcrosses eliminating maladaptive genotypic combinations. That we consistently find the same genes and developmental processes associated with restricted introgression in both transects may suggest that the mechanisms of selection against hybrids overlap over a broad geographic scale. However, the fact that the overwhelming majority of introgression-resistant genes correspond to the putative inversion on the Z chromosome makes it impossible to eliminate the alternate possibility that this signal is primarily driven by their location on a structural variant with little to no recombination. While the narrower cline in genome-wide ancestry in Missouri relative to Pennsylvania is consistent with stronger selection (Harrison & Larson, 2014), a secondary peak in the parameter density for this cline (Supplementary Figure S2) contributes to uncertainty in this estimate. Indeed, our sampling across the two transects differed substantially, with a less linear, more “mosaic” distribution of localities in Missouri (Figure 1). We therefore interpret our findings with caution, suggesting that the cline width might be broadly concordant over a large geographic scale but emphasizing the need for additional sampling to more rigorously test this hypothesis.

The observed difference in the rate of hybrid zone movement between central and eastern North America is likely linked to the continent-wide differences in the velocity of climate change (Alexander et al., 2022). We believe this is a plausible mechanism for variation in the frequency, spatial distribution, and outcomes of hybridization and introgression in chickadees. First, more slowly moving hybrid zones should permit hybridization and backcrossing over a greater number of generations, hence providing more opportunity for bi- or unidirectional introgression. In turn, introgressed maladaptive alleles are more likely to be exposed to local selection pressures, as meiosis and recombination will generate novel genotypes, allowing neutral or beneficial variants to more freely diffuse across the tension zone. Our simulations of hybrid zone dynamics and analysis of climatic variation indicate that climate-driven variation in the rate of a hybrid zone movement can contribute to genome-wide introgression patterns to some extent. Nonetheless, we point out that overall similarity of genome-wide hybridization and introgression patterns (outside of Z-linked inversion) suggests limited genomic footprints of these mechanism.

Of the three climatic variables differing the most between transects (mean temperature of the coldest quarter, precipitation of the coldest quarter, mean diurnal range, see Supplementary Results), all indicated more severe climatic conditions in Pennsylvania, which we hypothesize has a profound impact on chickadee overwinter survival. Previous studies have indicated that extreme winter conditions such as cold snaps, snowstorms, and extended periods of cold weather are strong mortality factors in many small passerines and for chickadees in particular (Petit et al., 2017). In turn, black-capped and Carolina chickadee hybrids have reduced metabolic efficiency (i.e., reduced metabolic scope), which makes them more vulnerable to severe weather than either parental species (Olson et al., 2010). Previous research suggesting that hybrid chickadees have a reduced ability to recover cached food sources (McQuillan et al., 2018) due to learning and cognitive deficiencies—most likely leading to mortality during the winter—adds evidence to an overall picture of stronger climate-driven selection in Pennsylvania. Interestingly, genes underlying aerobic metabolic processes and neurological functions are broadly interconnected (Supplementary Figure S3), indicating that biological functions may be tightly linked on an organismal level. It therefore appears plausible that climatic differences between Missouri and Pennsylvania result in stronger selection against hybrids in the latter; however, we did not find strong support for this in genome-wide introgression patterns, proportion of distinct hybrid classes, or width of the hybrid zone. We note that comparatively stronger selection in Pennsylvania could still result in reduced locus-specific introgression of genes relevant to phenotypic aspects of hybrid disadvantage (e.g., basal metabolism and spatial cognition). Exploring this possibility will require trait-specific genome-wide association studies and documenting introgression patterns of trait-linked loci.

In spite of variation in climate and the rate of hybrid zone movement in Missouri and Pennsylvania, only the Z chromosome appears to show major differences between transects. Numerous previous studies have shown that the sex chromosomes typically have more restricted introgression relative to autosomes (including in chickadees, e.g., Taylor, Curry, et al., 2014). The mechanisms behind differences in introgression rates between sex chromosomes and autosomes vary, including suppressed recombination, smaller effective population size, and faster lineage sorting (Bachtrog, 2013; Charlesworth & Charlesworth, 2000), and a disproportionally higher fraction of genes involved in adaptation and speciation (Payseur et al., 2018). However, the specific mechanisms affecting introgression in the chickadee system are currently unknown, and the dramatic differences in Z-specific introgression rates between Missouri and Pennsylvania are intriguing. One possible explanation is that selection strength on Z-specific genes is relaxed in Missouri compared to Pennsylvania, a hypothesis consistent with observed differences in climate between states. However, an explicit connection between Z-linked genes and local environmental conditions remains elusive.

Another hypothesis for elevated differentiation on the Z chromosome concerns its putative chromosomal inversions. It is striking that the only genomic region maintaining strong differences across transects in relative differentiation and levels of gene flow is the right arm of the Z chromosome—specifically, the haplotype restricted to eastern Carolina chickadees. While this claim is impossible to assess without long read data, a plausible scenario is that the eastern Carolina Z haplotype is a product of *two* inversions: one between black-capped and Carolina chickadees and another between Carolina chickadee populations. If present, these inversions would create substantial rearrangement in gene order and overall Z architecture and plausibly be a major source of partial reproductive isolation between black-capped and Carolina chickadees. Previous studies have identified structural variants as a prominent source of lower fitness in hybrids through multiple mechanisms (reviewed in Hooper & Price, 2017; Kirkpatrick & Barton, 2006; Hoffmann & Rieseberg, 2008; Zhang et al., 2021, but see Westram et al., 2021). This could be directly relevant for the observed reduced hatching success in admixed chickadees (Bronson, Grubb, Sattler et al., 2003, 2005; Driver et al., 2022) and appears to underlie patterns of reduced introgression across multiple hybrid zone transects.

Broadly, the results of our study suggest that similar selective pressures may shape patterns of hybridization and introgression across the vast contact zone between black-capped and Carolina chickadees. Moreover, the genetic basis of traits under selection appears similar based on similar patterns of introgression. This adds evidence to an emerging consensus that certain genes, genomic regions, and the aspects of phenotype that they are associated with, may be disproportionally involved in divergent evolution within and between lineages (Baiz et al., 2021; Chen et al., 2013; Jones et al., 2012). Observed differences in introgression rates, especially prominent on the Z chromosome, could be driven by differences in the strength of selection and the rate of hybrid zone movement itself. However, our results also highlight the possibility of large-scale inversions on the Z chromosome which, if confirmed, are likely (if not the primary) contributors to reduced Z-specific introgression rates between chickadee species. These findings agree with those from other studies that suggest a major role for sex chromosomes in maintaining species boundaries and promoting speciation (Payseur et al., 2018). Furthermore, several studies have highlighted the role of inversions on the Z chromosome as a factor promoting rapid and strong reproductive isolation (Hooper & Price, 2017). Putative Z chromosome inversions between chickadee populations and species thus add to an emerging pattern wherein sex chromosome inversions play a prominent role in avian differentiation. Recently, genetic regions crucial for conspecific and heterospecific identification and sexually selected plumage markers were found within Z chromosome inversions in Baltimore and Bullock’s orioles (Walsh et al., 2023) and common yellowthroats (Dunn et al., 2024), respectively. Beyond plumage traits, Z chromosome inversions have been associated with sperm morphology in zebra finches (Knief et al., 2017) and differentiation in insects (Sharakhov & Sharakhova, 2024). Z chromosome inversions may be playing a major role in maintaining reproductive barriers between black-capped and Carolina chickadees, but this should be validated using long-read sequencing data. The complete geographic sorting of the Z chromosome types we observed suggests that there might be partial or even complete reproductive isolation between eastern and western Carolina chickadee lineages, which should be further studied through an additional hybrid zone analysis at their point of contact.

## Supplementary material

Supplementary material is available online at *Evolution Letters*.

qraf009_suppl_Supplementary_Tables_S1_Figures_S1-S5

## Data Availability

Revant code and data files are available on Dryad https://doi.org/10.5061/dryad.fn2z34v68 and GitHub (https://github.com/erikrfunk/Chickadee_moving-hybrid-zone_simulations). Any additional information and data can be obtained from the authors by request.
