# Supplementary material for: Replicate geographic transects across a hybrid zone reveal parallelism and differences in the genetic architecture of reproductive isolation
Source: Evol Lett. 2025 May 7;9(4):421–33. doi: 10.1093/evlett/qraf009 (PMC12448226; doi:10.1093/evlett/qraf009)
Supplement: qraf009_suppl_Supplementary_Tables_S1_Figures_S1-S5 [file qraf009_suppl_supplementary_tables_s1_figures_s1-s5.pdf]

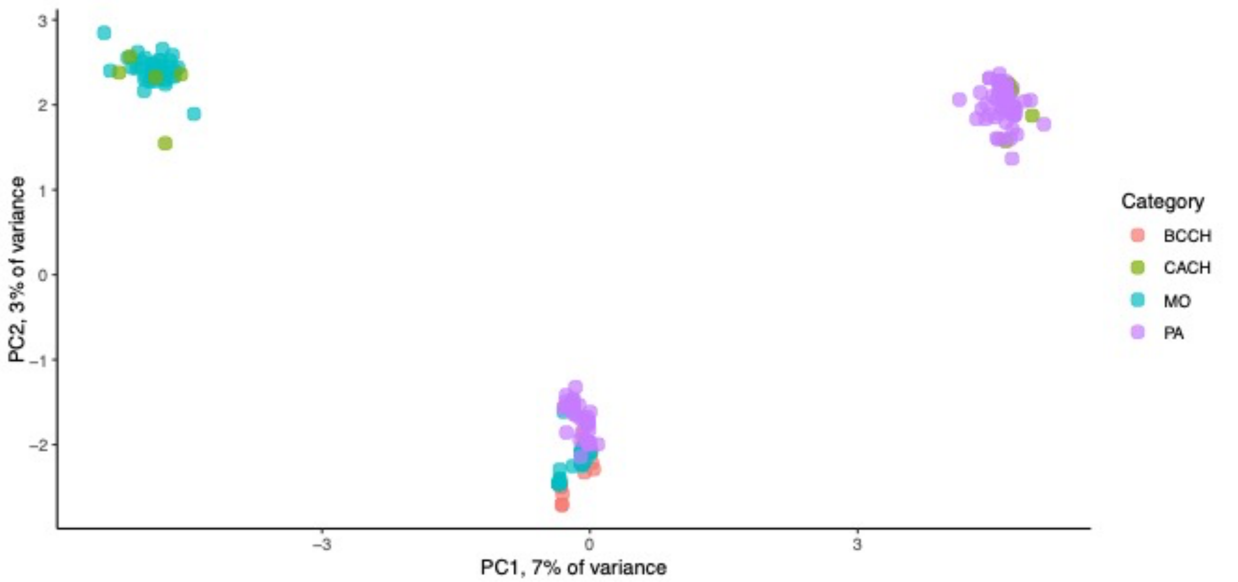

Figure S1. Results of Principal Component Analysis using SNPs from the mitochondrial genome (n=212) in allopatric Black-capped (pink) and Carolina (green) chickadees and from the transects through their hybrid zone in Missouri (MO, blue) and Pennsylvania (PA, purple).

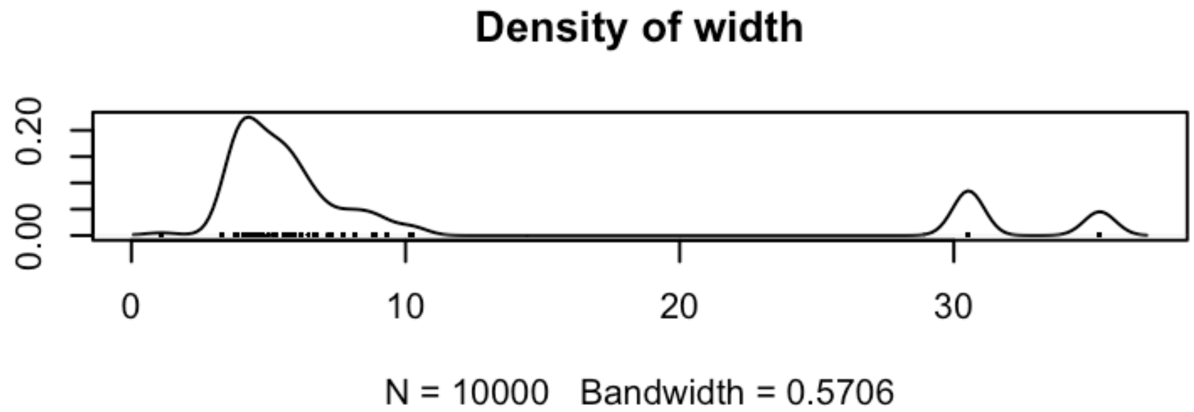

Figure S2. Density distribution for “width” parameter for the geographic cline of the ancestry transition in the Missouri transect.

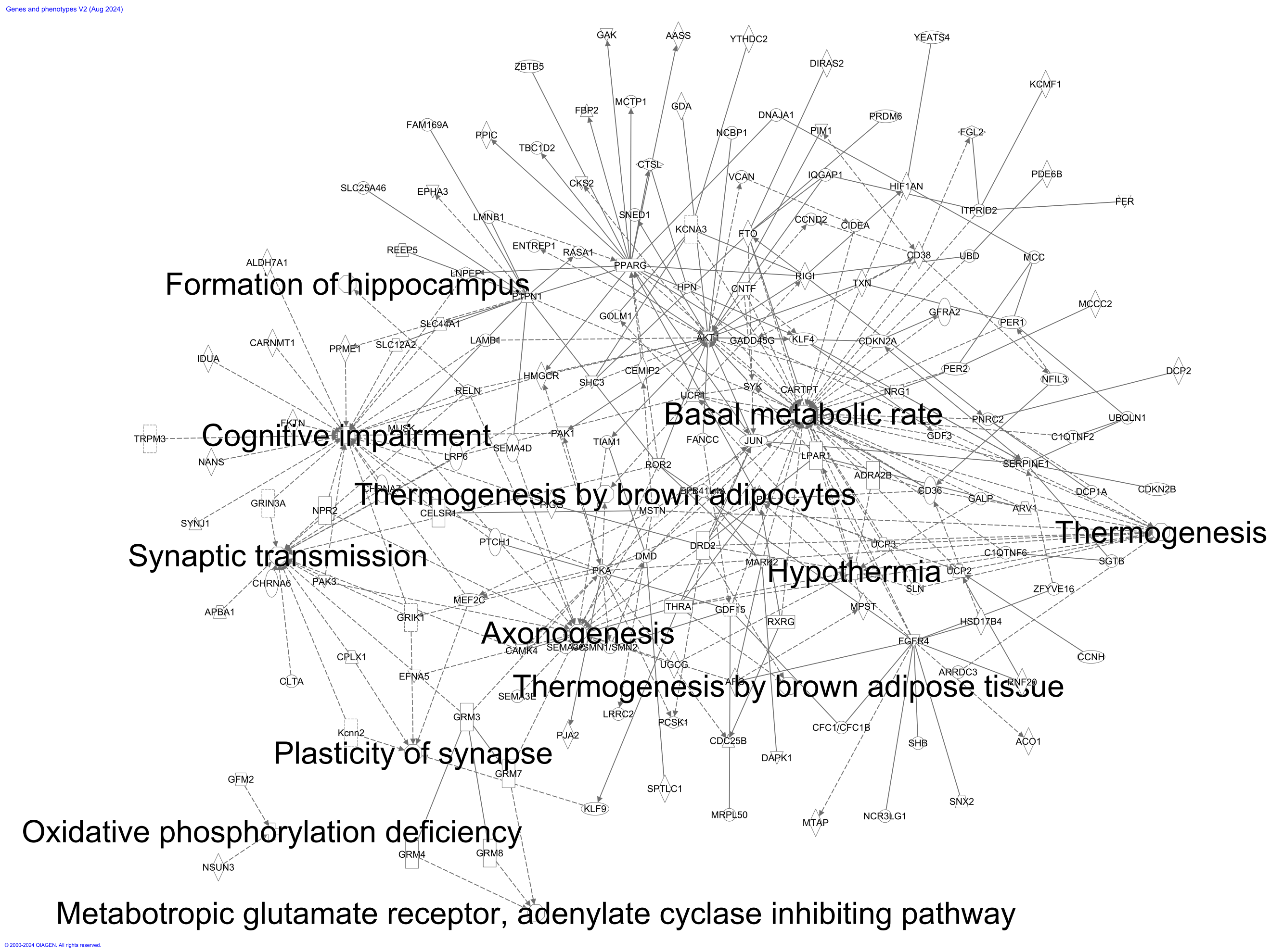

Figure S3. Genes with restricted introgression in both Missouri and Pennsylvania transects, and corresponding phenotypes known to be affected by these genes as identified by the Ingenuity Pathway Analysis.

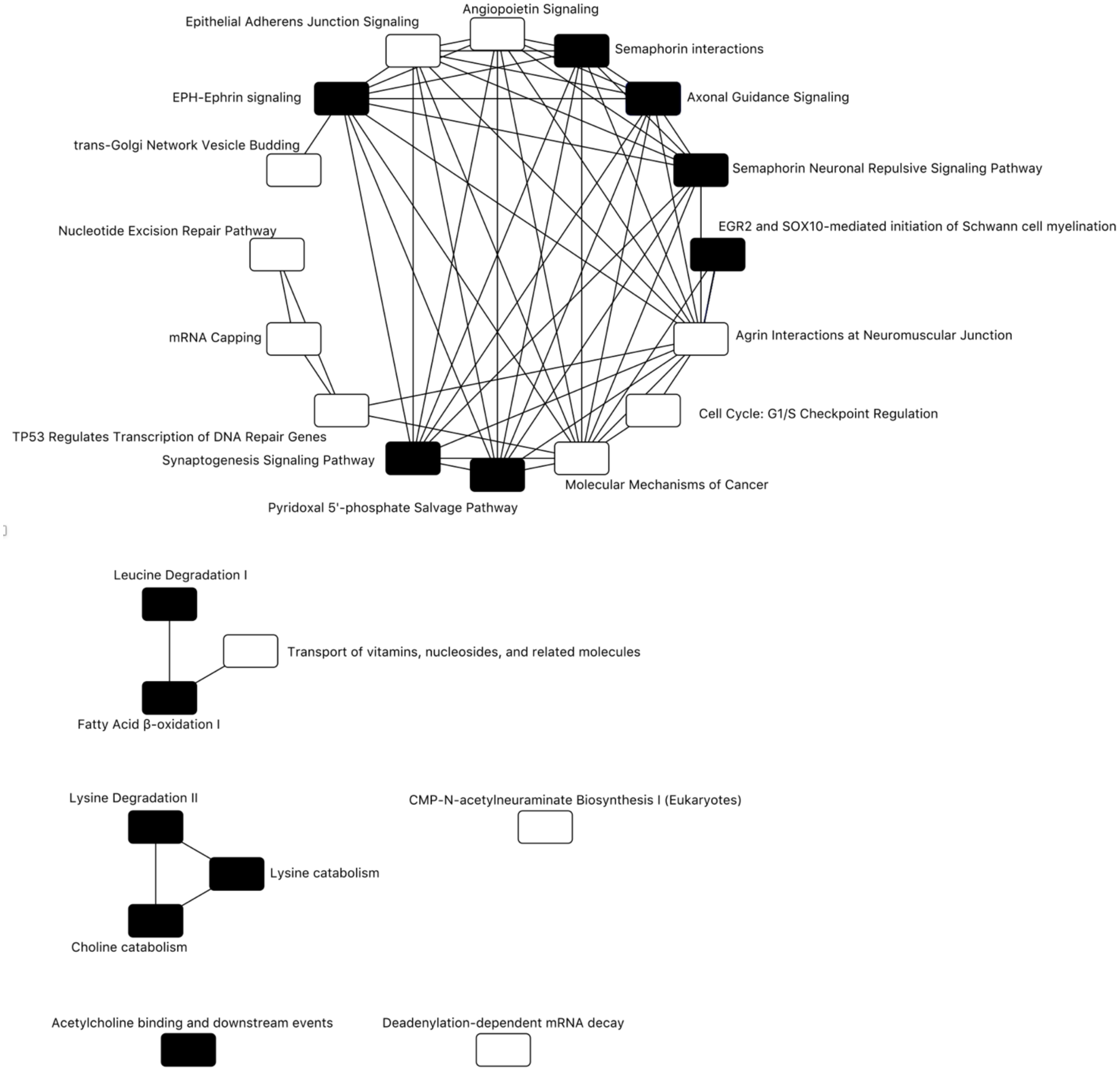

Figure S4. Biological pathways with significant overrepresentation of genes with introgression restricted in both Missouri and Pennsylvania transects. Black filling indicates pathways with apparent connection to physiological and neurological functions, which both are known targets of selections in black-capped and Carolina chickadee hybrid zone.

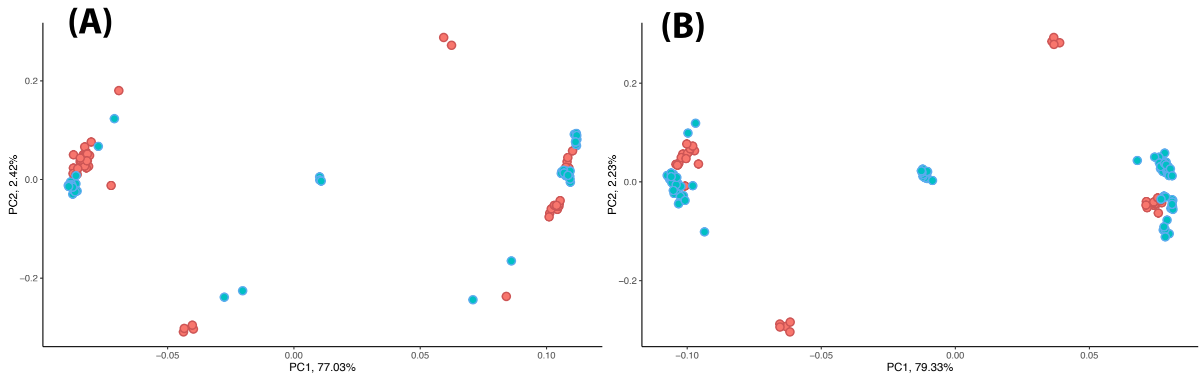

Figure S5. Results of Principal Component Analysis showing haplotypic segregation for inverted regions on the Z chromosome (SNPs within the boundaries of the putative inversion) in Pennsylvania (A) and Missouri (B). Red and blue dots correspond to females and males respectively. Inversion has slightly different end boundary in the two transects, from 4,019,600 to 139,615,224 bp in Pennsylvania, and from 4,019,600 to 137,849,599 bp in Missouri. Note that the spread of PC2 values groups males and females that are heterozygous for the inverted genotype in the way that may suggest limited recombination between haplotypes, e.g., in the pseudo-autosomal region of the Z. This hypothesis should be further addressed using long read sequencing data.

Supplementary tables:

Table S1: Sampling details and associated information

ID of vcf file	Transect/status	Field assigned sex	Collection site	Latitude	Longitude	Collection date	Grouping for transects	Distance along transect, km	MtDNA type
2830_31820_S1_L003	Allopatric BCCH	F	Shopnitz's Backyard	40.05	-105.27	7/13/18	Boulder	0	BCCH
2830_31836_S3_L003	Allopatric BCCH	M	2868 Loma Pl	40.05	-105.27	6/6/18	Boulder	0	BCCH
2830_31843_S72_L003	Allopatric BCCH	F	Shopnitz's Backyard	40.05	-105.27	6/13/18	Boulder	0	BCCH
2830_31844_S4_L003	Allopatric BCCH	M	Deane's House	40.05	-105.27	6/14/18	Boulder	0	BCCH
2830_31845_S14_L003	Allopatric BCCH	F	Deane's House	40.05	-105.27	6/14/18	Boulder	0	BCCH
2830_31846_S24_L003	Allopatric BCCH	M	Deane's House	40.05	-105.27	6/14/18	Boulder	0	BCCH
2830_31847_S34_L003	Allopatric BCCH	M	Kendi's House	40.05	-105.27	6/21/18	Boulder	0	BCCH
2830_31848_S44_L003	Allopatric BCCH	M	Kendi's House	40.05	-105.27	6/21/18	Boulder	0	BCCH
2830_31849_S54_L003	Allopatric BCCH	F	Nathan's House	40.05	-105.27	6/22/18	Boulder	0	BCCH
2830_31850_S64_L003	Allopatric BCCH	M	Nathan's House	40.05	-105.27	6/22/18	Boulder	0	BCCH
2830_31852_S5_L003	Allopatric BCCH	F	Nathan's House	40.05	-105.27	6/22/18	Boulder	0	BCCH
2830_31854_S25_L003	Allopatric BCCH	M	KK's House	40.05	-105.27	6/28/18	Boulder	0	BCCH
2830_31855_S35_L003	Allopatric BCCH	F	Nathan's House	40.05	-105.27	6/29/18	Boulder	0	BCCH
2830_31859_S74_L003	Allopatric BCCH	F	Norris's House	40.05	-105.27	7/5/18	Boulder	0	BCCH
2830_31862_S26_L003	Allopatric BCCH	F	Shopnitz's Backyard	40.05	-105.27	7/24/18	Boulder	0	BCCH
A32611	MO	F	north side of Butler Lake, Miami Creek	38.28	-94.43	4/13/16	Boulder	951	BCCH
A32612	MO	M	north side of Butler Lake, Miami Creek	38.28	-94.43	4/13/16	Boulder	951	BCCH
A32613	MO	F	north side of Butler Lake, Miami Creek	38.28	-94.43	4/13/16	Boulder	951	BCCH
A32614	MO	M	north side of Butler Lake, Miami Creek	38.28	-94.43	4/13/16	Boulder	951	NA
A32615	MO	M	north side of Butler Lake, Miami Creek	38.28	-94.43	4/13/16	Boulder	951	BCCH
A32616	MO	F	north side of Butler Lake, Miami Creek	38.28	-94.43	4/13/16	Boulder	951	BCCH
A32617	MO	M	north side of Butler Lake, Miami Creek	38.28	-94.43	4/13/16	Boulder	951	BCCH

A35820	MO	F	Settle's Ford Conservation Area	38.44	-94.13	3/17/19	Settle's Ford	956	BCCH
A35821	MO	M	Settle's Ford Conservation Area	38.44	-94.13	3/17/19	Settle's Ford	956	BCCH
A35823	MO	M	Settle's Ford Conservation Area	38.44	-94.13	3/17/19	Settle's Ford	956	BCCH
A35826	MO	F	Settle's Ford Conservation Area	38.44	-94.13	3/17/19	Settle's Ford	956	BCCH
A35827	MO	M	Settle's Ford Conservation Area	38.44	-94.13	3/17/19	Settle's Ford	956	BCCH
A35841	MO	M	Settle's Ford Conservation Area	38.44	-94.13	3/26/19	Settle's Ford	956	BCCH
A35849	MO	M	Settle's Ford Conservation Area	38.44	-94.13	3/26/19	Settle's Ford	956	BCCH
A35865	MO	M	Settle's Ford Conservation Area	38.44	-94.13	4/9/19	Settle's Ford	956	BCCH
A34813	MO	F	ca. 2.5 miles northeast of Rich Hill	38.16	-94.33	4/26/18	Peabody	967	BCCH
A34814	MO	M	ca. 2.5 miles northeast of Rich Hill	38.16	-94.33	4/26/18	Peabody	967	BCCH
A34815	MO	M	ca. 1.5 miles northeast of Rich Hill	38.16	-94.33	4/26/18	Peabody	967	BCCH
A34818	MO	M	ca. 3 miles northeast of Rich Hill	38.16	-94.33	4/26/18	Peabody	967	BCCH
A34819	MO	M	ca. 2.5 miles northeast of Rich Hill	38.16	-94.33	4/26/18	Peabody	967	BCCH
A34821	MO	M	Peabody Conservation Area	38.09	-94.44	4/20/18	Peabody	967	BCCH
A34822	MO	F	Peabody Conservation Area	38.09	-94.44	4/20/18	Peabody	967	BCCH
A34823	MO	M	Peabody Conservation Area	38.09	-94.44	4/20/18	Peabody	967	BCCH
A34824	MO	F	Peabody Conservation Area	38.09	-94.44	4/20/18	Peabody	967	BCCH
A34825	MO	F	Peabody Conservation Area	38.09	-94.44	4/20/18	Peabody	967	BCCH
A34826	MO	M	Peabody Conservation Area	38.09	-94.44	4/20/18	Peabody	967	BCCH
A34827	MO	M	Peabody Conservation Area	38.09	-94.44	4/20/18	Peabody	967	BCCH
A34828	MO	M	Peabody Conservation Area	38.09	-94.44	4/20/18	Peabody	967	BCCH
A34829	MO	M	Peabody Conservation Area	38.09	-94.44	4/20/18	Peabody	967	BCCH
A35840	MO	M	Rich Hill, ca. 5 miles NNE, Miami Creek Rich Hill,	38.16	-94.33	3/26/19	Peabody	967	BCCH
A35850	MO	F	Rich Hill, ca. 5 miles NNE, Miami Creek Drainage	38.16	-94.33	3/26/19	Peabody	967	BCCH
A35859	MO	F	Rich Hill, north of	38.16	-94.33	4/9/19	Peabody	967	BCCH
A35861	MO	M	Rich Hill, north of	38.16	-94.33	4/9/19	Peabody	967	BCCH
A35864	MO	F	Rich Hill, north of	38.16	-94.33	4/9/19	Peabody	967	BCCH
A35866	MO	M	Rich Hill, north of	38.16	-94.33	4/9/19	Peabody	967	BCCH

A32591	MO	M	Pleasant Gap, west of	38.17	-94.19	4/28/16	Pleasant Gap	974	BCCH
A32592	MO	F	Pleasant Gap, west of	38.17	-94.19	4/28/16	Pleasant Gap	974	CACH
A32593	MO	M	Pleasant Gap, west of	38.17	-94.19	4/28/16	Pleasant Gap	974	BCCH
A32594	MO	M	Pleasant Gap, west of	38.17	-94.19	4/28/16	Pleasant Gap	974	BCCH
A32595	MO	M	Pleasant Gap, west of	38.17	-94.19	4/28/16	Pleasant Gap	974	BCCH
A32596	MO	F	Pleasant Gap, west of	38.17	-94.19	4/28/16	Pleasant Gap	974	BCCH
A32607	MO	F	Pleasant Gap, west of	38.17	-94.19	4/28/16	Pleasant Gap	974	BCCH
A32608	MO	M	Pleasant Gap, west of	38.17	-94.19	4/28/16	Pleasant Gap	974	CACH
A35814	MO	M	Poague Conservation Area	38.42	-93.85	3/17/19	Pleasant Gap	974	BCCH
A35819	MO	F	Poague Conservation Area	38.42	-93.85	3/17/19	Pleasant Gap	974	BCCH
A35828	MO	M	Poague Conservation Area	38.42	-93.85	3/17/19	Pleasant Gap	974	BCCH
A35833	MO	F	Poague Conservation Area	38.42	-93.85	3/26/19	Pleasant Gap	974	BCCH
A35844	MO	F	Poague Conservation Area	38.42	-93.85	3/26/19	Pleasant Gap	974	BCCH
A35845	MO	M	Poague Conservation Area	38.42	-93.85	3/26/19	Pleasant Gap	974	BCCH
A35852	MO	M	Poague Conservation Area	38.42	-93.85	4/9/19	Pleasant Gap	974	BCCH
A35862	MO	F	Poague Conservation Area	38.42	-93.85	4/9/19	Pleasant Gap	974	BCCH
A29902	MO	M	Montrose Conservation Area	38.31	-93.97	4/28/17	Montrose	976	BCCH
A29903	MO	M	Montrose Conservation Area	38.31	-93.97	4/28/17	Montrose	976	BCCH
A29905	MO	F	Montrose Conservation Area	38.31	-93.97	4/28/17	Montrose	976	BCCH
A34812	MO	M	Montrose Conservation Area	38.31	-93.97	4/26/18	Montrose	976	BCCH
A34816	MO	M	Montrose Conservation Area	38.31	-93.97	4/26/18	Montrose	976	BCCH
A34817	MO	F	Montrose Conservation Area	38.31	-93.97	4/26/18	Montrose	976	BCCH
A34820	MO	M	Montrose Conservation Area	38.31	-93.97	4/26/18	Montrose	976	BCCH
A32535	MO	NA	Four Rivers Conservation Area, east side of Unit 4	38.04	-94.25	4/13/16	Four Rivers	981	CACH
A32538	MO	M	Four Rivers Conservation Area, Unit 1, Prairie tract	38.04	-94.25	4/13/16	Four Rivers	981	BCCH
A32539	MO	M	Four Rivers Conservation Area, east side of Unit 2	38.04	-94.25	4/13/16	Four Rivers	981	BCCH
A32540	MO	F	Four Rivers Conservation Area, east side of Unit 4	38.04	-94.25	4/13/16	Four Rivers	981	BCCH
A32547	MO	F	Four Rivers Conservation Area, east side of Unit 4	38.04	-94.25	4/13/16	Four Rivers	981	BCCH

A32548	MO	M	Four Rivers Conservation Area, east side of Unit 4	38.04	-94.25	4/13/16	Four Rivers	981	BCCH
A32549	MO	M	Four Rivers Conservation Area, east side of Unit 4	38.04	-94.25	4/13/16	Four Rivers	981	CACH
A32550	MO	F	Four Rivers Conservation Area, east side of Unit 4	38.04	-94.25	4/13/16	Four Rivers	981	BCCH
A32551	MO	M	Four Rivers Conservation Area, east side of Unit 2, pool 12	38.04	-94.25	4/12/16	Four Rivers	981	BCCH
A32552	MO	F	Four Rivers Conservation Area, east side of Unit 2, pool 12	38.04	-94.25	4/12/16	Four Rivers	981	BCCH
A32597	MO	M	Four Rivers Conservation Area, south end of Unit 1	38.04	-94.25	4/13/16	Four Rivers	981	BCCH
A32534	MO	M	Appleton City, 1.7 miles southeast	38.17	-94.02	4/12/16	Rockville	985	CACH
A32536	MO	M	Appleton City, 1.7 miles southeast	38.17	-94.02	4/12/16	Rockville	985	CACH
A32537	MO	M	Appleton City, 2.2 miles southeast	38.17	-94.02	4/12/16	Rockville	985	CACH
A32541	MO	M	Rockville, 5.8 miles west (west of Prairie City)	38.11	-94.09	4/12/16	Rockville	985	CACH
A32542	MO	F	Rockville, 5.8 miles west (west of Prairie City)	38.11	-94.09	4/12/16	Rockville	985	CACH
A32543	MO	M	Appleton City, 1.7 miles southeast	38.17	-94.02	4/12/16	Rockville	985	CACH
A32545	MO	F	Appleton City Cemetery, adjacent to east side	38.17	-94.02	4/12/16	Rockville	985	CACH
A32546	MO	M	Appleton City Cemetery, adjacent to east side	38.17	-94.02	4/12/16	Rockville	985	CACH
A32553	MO	M	Rockville, 3.2 miles north	38.10	-94.10	4/9/16	Rockville	985	CACH
A32554	MO	M	Appleton City Cemetery	38.17	-94.02	4/9/16	Rockville	985	BCCH
A32555	MO	F	Rockville, 3.2 miles north	38.10	-94.10	4/9/16	Rockville	985	BCCH
A32556	MO	M	Rockville, 3.2 miles north	38.09	-94.10	4/9/16	Rockville	985	BCCH
A32557	MO	F	Rockville, 1.5 miles southeast	38.09	-94.10	4/8/16	Rockville	985	CACH
A32558	MO	M	Rockville, 1.5 miles southeast	38.09	-94.10	4/8/16	Rockville	985	CACH
A32559	MO	M	Rockville, 3.2 miles north	38.09	-94.11	4/9/16	Rockville	985	CACH
A32560	MO	F	Rockville, 3.2 miles north	38.09	-94.11	4/9/16	Rockville	985	CACH
A32567	MO	M	Rockville, 2.3 miles west	38.08	-94.11	4/8/16	Rockville	985	BCCH
A32568	MO	M	Rockville, 3.2 miles north	38.08	-94.11	4/9/16	Rockville	985	BCCH
A32569	MO	M	Rockville, 1.5 miles southeast	38.08	-94.11	4/8/16	Rockville	985	CACH
A32570	MO	F	Rockville, 1.5 miles southeast	38.08	-94.11	4/8/16	Rockville	985	CACH
A32571	MO	M	Rockville, 4 miles northwest	38.08	-94.11	4/9/16	Rockville	985	BCCH
A32572	MO	F	Rockville, 4.1 miles west (east of Prairie City)	38.07	-94.11	4/8/16	Rockville	985	CACH

A32573	MO	M	Rockville, 4.1 miles west (east of Prairie City)	38.07	-94.11	4/8/16	Rockville	985	BCCH
A32574	MO	M	Rockville, 4 miles northwest	38.07	-94.11	4/9/16	Rockville	985	BCCH
A32575	MO	F	Rockville, 4.1 miles west (east of Prairie City)	38.06	-94.09	4/8/16	Rockville	985	CACH
A32576	MO	M	Rockville, 4.1 miles west (east of Prairie City)	38.06	-94.08	4/8/16	Rockville	985	CACH
A32577	MO	M	Rockville, 4.1 miles west (east of Prairie City)	38.05	-94.06	4/8/16	Rockville	985	BCCH
A32578	MO	M	Rockville, 5.8 miles west (west of Prairie City)	38.04	-94.05	4/8/16	Rockville	985	NA
A32579	MO	F	Rockville, 5.8 miles west (west of Prairie City)	38.04	-94.03	4/8/16	Rockville	985	NA
A32580	MO	M	Rockville, 1.5 miles southeast	38.03	-94.02	4/8/16	Rockville	985	BCCH
A32581	MO	M	Appleton City, 1.7 miles southeast	38.17	-94.02	4/9/16	Rockville	985	CACH
A32582	MO	M	Appleton City Cemetery	38.17	-94.02	4/9/16	Rockville	985	CACH
A32583	MO	F	Appleton City Cemetery	38.17	-94.02	4/9/16	Rockville	985	CACH
A32584	MO	M	Rockville, 2.3 miles west	37.74	-93.89	4/8/16	Rockville	985	CACH
A32585	MO	M	Rockville, 3.2 miles north	37.49	-93.79	4/9/16	Rockville	985	CACH
A32586	MO	F	Rockville, 3.2 miles north	37.23	-93.70	4/9/16	Rockville	985	BCCH
A32587	MO	M	Rockville, 2.3 miles west	36.98	-93.60	4/8/16	Rockville	985	BCCH
A32588	MO	F	Rockville, 2.3 miles west	36.72	-93.50	4/8/16	Rockville	985	CACH
A32589	MO	M	Rockville, 4 miles northwest	36.64	-92.95	4/9/16	Rockville	985	CACH
A32590	MO	F	Rockville, 4 miles northwest	36.56	-92.39	4/9/16	Rockville	985	CACH
A32561	MO	F	Schell-Osage Conservation Area, southwest section	38.03	-94.10	4/5/16	Schell-Osage	991	CACH
A32562	MO	M	Schell-Osage Conservation Area, southwest section	38.03	-94.10	4/5/16	Schell-Osage	991	CACH
A32563	MO	M	Schell-Osage Conservation Area, southwest section	38.03	-94.10	4/5/16	Schell-Osage	991	CACH
A32564	MO	F	Schell-Osage Conservation Area, southwest section	38.03	-94.10	4/5/16	Schell-Osage	991	CACH
A32598	MO	F	Schell-Osage Conservation Area, northwest section	38.03	-94.10	4/5/16	Schell-Osage	991	CACH
A32599	MO	M	Schell-Osage Conservation Area, northeast section	38.03	-94.10	4/4/16	Schell-Osage	991	CACH
A32600	MO	F	Schell-Osage Conservation Area, northeast section	38.03	-94.10	4/4/16	Schell-Osage	991	CACH
A32603	MO	M	Schell-Osage Conservation Area, northwest section	38.03	-94.10	4/5/16	Schell-Osage	991	CACH
A32604	MO	M	Schell-Osage Conservation Area, northwest section	38.03	-94.10	4/5/16	Schell-Osage	991	CACH
A32605	MO	M	Schell-Osage Conservation Area, southwest section	38.03	-94.10	4/5/16	Schell-Osage	991	CACH

A32606	MO	F	Schell-Osage Conservation Area, southwest section	38.03	-94.10	4/5/16	Schell-Osage	991	CACH
A32609	MO	F	Schell-Osage Conservation Area, northwest section	38.03	-94.10	4/5/16	Schell-Osage	991	CACH
A32610	MO	F	Schell-Osage Conservation Area, northwest section	38.03	-94.10	4/5/16	Schell-Osage	991	CACH
A32618	MO	M	Bird Song Conservation Area	37.88	-93.71	4/4/16	Bird Song	1026	CACH
A32619	MO	M	Bird Song Conservation Area	37.88	-93.71	4/4/16	Bird Song	1026	CACH
A32620	MO	F	Bird Song Conservation Area	37.88	-93.71	4/4/16	Bird Song	1026	CACH
A32621	MO	F	Bird Song Conservation Area	37.88	-93.71	4/4/16	Bird Song	1026	CACH
A32622	MO	M	Bird Song Conservation Area	37.88	-93.71	4/4/16	Bird Song	1026	CACH
A32623	MO	M	Bird Song Conservation Area	37.88	-93.71	4/4/16	Bird Song	1026	CACH
A32624	MO	M	Bird Song Conservation Area	37.88	-93.71	4/4/16	Bird Song	1026	CACH
A32625	MO	M	Bird Song Conservation Area	37.88	-93.71	4/4/16	Bird Song	1026	CACH
A32626	MO	M	Bird Song Conservation Area	37.88	-93.71	4/4/16	Bird Song	1026	CACH
S_77292	Western CACH	NA	Baton Rouge, Louisiana	30.41	-91.18	NA	Louisiana	1805	CACH
S_77293	Western CACH	NA	Baton Rouge, Louisiana	30.41	-91.18	NA	Louisiana	1805	CACH
S_77294	Western CACH	NA	Baton Rouge, Louisiana	30.41	-91.18	NA	Louisiana	1805	CACH
S_77296	Western CACH	NA	Baton Rouge, Louisiana	30.41	-91.18	NA	Louisiana	1805	CACH
S_77297	Western CACH	NA	Baton Rouge, Louisiana	30.41	-91.18	NA	Louisiana	1805	CACH
CACH32_1	Eastern CACH	F	East Carolina University, North Carolina	35.63	-77.49	4/28/19	North Carolina	2420	CACH
CACH5_1	Eastern CACH	F	East Carolina University, North Carolina	35.63	-77.49	5/4/19	North Carolina	2420	CACH
E1032	Eastern CACH	NA	East Carolina University, North Carolina	35.63	-77.49	3/30/19	North Carolina	2420	CACH
E1033	Eastern CACH	M	East Carolina University, North Carolina	35.63	-77.49	3/31/19	North Carolina	2420	CACH
E1034	Eastern CACH	F	East Carolina University, North Carolina	35.63	-77.49	3/31/19	North Carolina	2420	CACH
E1035	Eastern CACH	M	East Carolina University, North Carolina	35.63	-77.49	3/31/19	North Carolina	2420	CACH
B_25801	PA	M	DeRuyter, New York	42.76	-75.89	5/27/19	New York	2526	BCCH
B_25902	PA	F	DeRuyter, New York	42.76	-75.89	5/27/19	New York	2526	BCCH
B_25903	PA	M	DeRuyter, New York	42.76	-75.89	5/27/19	New York	2526	BCCH
B_25904	PA	M	DeRuyter, New York	42.76	-75.89	5/27/19	New York	2526	BCCH
BCCH10	PA	NA	Ithaca, New York	42.44	-76.50	NA	Ithaca	2536	BCCH

BCCH1A	PA	NA	Ithaca, New York	42.44	-76.50	NA	Ithaca	2536	BCCH
BCCH2	PA	NA	Ithaca, New York	42.44	-76.50	NA	Ithaca	2536	BCCH
BCCH30	PA	NA	Ithaca, New York	42.44	-76.50	NA	Ithaca	2536	BCCH
BCCH4	PA	NA	Ithaca, New York	42.44	-76.50	NA	Ithaca	2536	BCCH
BCCH5	PA	NA	Ithaca, New York	42.44	-76.50	NA	Ithaca	2536	BCCH
BCCH6	PA	NA	Ithaca, New York	42.44	-76.50	NA	Ithaca	2536	BCCH
BCCH7	PA	NA	Ithaca, New York	42.44	-76.50	NA	Ithaca	2536	BCCH
BCCH8	PA	NA	Ithaca, New York	42.44	-76.50	NA	Ithaca	2536	BCCH
E458	PA	M	Hickory Run State Park, Pennsylvania	41.03	-75.68	7/29/15	Hickory run	2705	BCCH
E461_merged	PA	NA	Hickory Run State Park, Pennsylvania	41.03	-75.68	NA	Hickory run	2705	BCCH
E463_merged	PA	NA	Hickory Run State Park, Pennsylvania	41.03	-75.68	NA	Hickory run	2705	BCCH
E464_merged	PA	NA	Hickory Run State Park, Pennsylvania	41.03	-75.68	NA	Hickory run	2705	BCCH
E466_merged	PA	NA	Hickory Run State Park, Pennsylvania	41.03	-75.68	NA	Hickory run	2705	BCCH
E635_merged	PA	NA	Hickory Run State Park, Pennsylvania	41.03	-75.68	NA	Hickory run	2705	BCCH
E639	PA	M	Hickory Run State Park, Pennsylvania	41.03	-75.68	6/4/16	Hickory run	2705	BCCH
E809_merged	PA	NA	Hickory Run State Park, Pennsylvania	41.03	-75.68	NA	Hickory run	2705	BCCH
E980	PA	M	Hickory Run State Park, Pennsylvania	41.03	-75.68	6/20/18	Hickory run	2705	BCCH
E983	PA	F	Hickory Run State Park, Pennsylvania	41.03	-75.68	6/20/18	Hickory run	2705	BCCH
E441_2	PA	M	Jacobsburg State Park, Pennsylvania	40.80	-75.31	5/25/16	Jacobsburg	2741	BCCH
E442	PA	F	Jacobsburg State Park, Pennsylvania	40.80	-75.31	7/22/15	Jacobsburg	2741	BCCH
E446	PA	M	Jacobsburg State Park, Pennsylvania	40.80	-75.31	7/23/15	Jacobsburg	2741	CACH
E447	PA	F	Jacobsburg State Park, Pennsylvania	40.80	-75.31	7/23/15	Jacobsburg	2741	BCCH
E451	PA	F	Jacobsburg State Park, Pennsylvania	40.80	-75.31	7/23/15	Jacobsburg	2741	CACH
E452	PA	F	Jacobsburg State Park, Pennsylvania	40.80	-75.31	7/23/15	Jacobsburg	2741	CACH
E453	PA	M	Jacobsburg State Park, Pennsylvania	40.80	-75.31	7/23/15	Jacobsburg	2741	CACH
E454_2	PA	M	Jacobsburg State Park, Pennsylvania	40.80	-75.31	6/10/16	Jacobsburg	2741	BCCH
E457	PA	F	Jacobsburg State Park, Pennsylvania	40.80	-75.31	7/29/15	Jacobsburg	2741	BCCH
E641	PA	M	Jacobsburg State Park, Pennsylvania	40.80	-75.31	6/10/16	Jacobsburg	2741	BCCH

E688	PA	F	Jacobsburg State Park, Pennsylvania	40.80	-75.31	5/25/16	Jacobsburg	2741	CACH
E696_merged	PA	NA	Jacobsburg State Park, Pennsylvania	40.80	-75.31	NA	Jacobsburg	2741	CACH
E698_merged	PA	NA	Jacobsburg State Park, Pennsylvania	40.80	-75.31	NA	Jacobsburg	2741	CACH
E699_merged	PA	NA	Jacobsburg State Park, Pennsylvania	40.80	-75.31	NA	Jacobsburg	2741	BCCH
E700_merged	PA	NA	Jacobsburg State Park, Pennsylvania	40.80	-75.31	NA	Jacobsburg	2741	CACH
E927	PA	M	Jacobsburg State Park, Pennsylvania	40.80	-75.31	6/2/18	Jacobsburg	2741	BCCH
E935	PA	F	Jacobsburg State Park, Pennsylvania	40.80	-75.31	6/4/18	Jacobsburg	2741	BCCH
E938	PA	F	Jacobsburg State Park, Pennsylvania	40.80	-75.31	6/2/18	Jacobsburg	2741	CACH
E941	PA	M	Jacobsburg State Park, Pennsylvania	40.80	-75.31	6/4/18	Jacobsburg	2741	BCCH
E942	PA	F	Jacobsburg State Park, Pennsylvania	40.80	-75.31	6/4/18	Jacobsburg	2741	BCCH
E975	PA	M	Jacobsburg State Park, Pennsylvania	40.80	-75.31	6/16/18	Jacobsburg	2741	BCCH
E990	PA	M	Jacobsburg State Park, Pennsylvania	40.80	-75.31	8/8/18	Jacobsburg	2741	CACH
E992	PA	F	Jacobsburg State Park, Pennsylvania	40.80	-75.31	8/21/18	Jacobsburg	2741	CACH
E012	PA	F	Lehigh University, Pennsylvania	40.60	-75.38	5/29/13	Lehigh	2758	CACH
E030_merged	PA	NA	Lehigh University, Pennsylvania	40.60	-75.38	NA	Lehigh	2758	NA
E031_merged	PA	NA	Lehigh University, Pennsylvania	40.60	-75.38	NA	Lehigh	2758	CACH
E035_merged	PA	NA	Lehigh University, Pennsylvania	40.60	-75.38	NA	Lehigh	2758	CACH
E039	PA	F	Lehigh University, Pennsylvania	40.60	-75.38	6/4/13	Lehigh	2758	CACH
E042	PA	F	Lehigh University, Pennsylvania	40.60	-75.38	5/6/13	Lehigh	2758	CACH
E060	PA	F	Lehigh University, Pennsylvania	40.60	-75.38	5/26/13	Lehigh	2758	CACH
E070	PA	F	Lehigh University, Pennsylvania	40.60	-75.38	5/27/13	Lehigh	2758	CACH
E076	PA	F	Lehigh University, Pennsylvania	40.60	-75.38	6/4/14	Lehigh	2758	CACH
E081	PA	M	Lehigh University, Pennsylvania	40.60	-75.38	5/30/13	Lehigh	2758	CACH
E082	PA	F	Lehigh University, Pennsylvania	40.60	-75.38	5/30/13	Lehigh	2758	CACH
E085_merged	PA	NA	Lehigh University, Pennsylvania	40.60	-75.38	NA	Lehigh	2758	CACH
E095	PA	M	Lehigh University, Pennsylvania	40.60	-75.38	6/4/13	Lehigh	2758	CACH
E104	PA	F	Lehigh University, Pennsylvania	40.60	-75.38	6/4/13	Lehigh	2758	CACH
E110_merged	PA	NA	Lehigh University, Pennsylvania	40.60	-75.38	NA	Lehigh	2758	CACH

E111	PA	F	Lehigh University, Pennsylvania	40.60	-75.38	6/12/13	Lehigh	2758	CACH
E128	PA	M	Lehigh University, Pennsylvania	40.60	-75.38	5/7/14	Lehigh	2758	CACH
E136	PA	M	Lehigh University, Pennsylvania	40.60	-75.38	5/22/14	Lehigh	2758	CACH
E137_merged	PA	NA	Lehigh University, Pennsylvania	40.60	-75.38	NA	Lehigh	2758	BCCH
E175_merged	PA	NA	Lehigh University, Pennsylvania	40.60	-75.38	NA	Lehigh	2758	CACH
E232	PA	F	Lehigh University, Pennsylvania	40.60	-75.38	6/22/14	Lehigh	2758	CACH
E240	PA	F	Lehigh University, Pennsylvania	40.60	-75.38	6/16/14	Lehigh	2758	CACH
E247	PA	M	Lehigh University, Pennsylvania	40.60	-75.38	6/8/14	Lehigh	2758	BCCH
E271	PA	F	Lehigh University, Pennsylvania	40.60	-75.38	6/8/14	Lehigh	2758	CACH
E304_merged	PA	NA	Lehigh University, Pennsylvania	40.60	-75.38	NA	Lehigh	2758	CACH
E328	PA	F	Lehigh University, Pennsylvania	40.60	-75.38	5/15/15	Lehigh	2758	CACH
E346_merged	PA	NA	Lehigh University, Pennsylvania	40.60	-75.38	NA	Lehigh	2758	BCCH
E350	PA	M	Lehigh University, Pennsylvania	40.60	-75.38	5/29/15	Lehigh	2758	CACH
E392	PA	F	Lehigh University, Pennsylvania	40.60	-75.38	5/17/15	Lehigh	2758	BCCH
E414	PA	M	Lehigh University, Pennsylvania	40.60	-75.38	6/5/15	Lehigh	2758	CACH
E415	PA	M	Lehigh University, Pennsylvania	40.60	-75.38	6/5/15	Lehigh	2758	CACH
E448	PA	M	Lehigh University, Pennsylvania	40.60	-75.38	7/21/15	Lehigh	2758	CACH
E450	PA	M	Lehigh University, Pennsylvania	40.60	-75.38	7/17/15	Lehigh	2758	CACH
E456_3	PA	F	Lehigh University, Pennsylvania	40.60	-75.38	5/31/16	Lehigh	2758	CACH
E533_merged	PA	NA	Lehigh University, Pennsylvania	40.60	-75.38	NA	Lehigh	2758	CACH
E592_merged	PA	NA	Lehigh University, Pennsylvania	40.60	-75.38	NA	Lehigh	2758	CACH
E790_merged	PA	NA	Lehigh University, Pennsylvania	40.60	-75.38	NA	Lehigh	2758	CACH
E816	PA	F	Lehigh University, Pennsylvania	40.60	-75.38	5/31/17	Lehigh	2758	CACH
E836	PA	M	Lehigh University, Pennsylvania	40.60	-75.38	5/31/17	Lehigh	2758	CACH
E924	PA	M	Lehigh University, Pennsylvania	40.60	-75.38	6/2/18	Lehigh	2758	CACH
E945	PA	F	Lehigh University, Pennsylvania	40.60	-75.38	6/2/18	Lehigh	2758	CACH
E958	PA	F	Lehigh University, Pennsylvania	40.60	-75.38	6/7/18	Lehigh	2758	CACH
E959	PA	M	Lehigh University, Pennsylvania	40.60	-75.38	6/7/18	Lehigh	2758	CACH

E986	PA	F	Lehigh University, Pennsylvania	40.60	-75.38	7/26/18	Lehigh	2758	CACH
E987	PA	M	Lehigh University, Pennsylvania	40.60	-75.38	7/27/18	Lehigh	2758	CACH
E054	PA	M	DeSales University, Pennsylvania	40.54	-75.38	5/22/13	DeSales	2765	CACH
E058	PA	M	DeSales University, Pennsylvania	40.54	-75.38	5/24/13	DeSales	2765	CACH
E132	PA	F	DeSales University, Pennsylvania	40.54	-75.38	5/7/14	DeSales	2765	CACH
E143	PA	M	DeSales University, Pennsylvania	40.54	-75.38	5/29/14	DeSales	2765	CACH
E162	PA	F	DeSales University, Pennsylvania	40.54	-75.38	5/29/14	DeSales	2765	CACH
E336_merged	PA	NA	DeSales University, Pennsylvania	40.54	-75.38	NA	DeSales	2765	CACH
E563	PA	F	DeSales University, Pennsylvania	40.54	-75.38	5/18/16	DeSales	2765	CACH
E564_merged	PA	NA	DeSales University, Pennsylvania	40.54	-75.38	NA	DeSales	2765	CACH
E578	PA	F	DeSales University, Pennsylvania	40.54	-75.38	5/23/16	DeSales	2765	CACH
E583_merged	PA	NA	DeSales University, Pennsylvania	40.54	-75.38	NA	DeSales	2765	CACH
E630_merged	PA	NA	DeSales University, Pennsylvania	40.54	-75.38	NA	DeSales	2765	CACH

SUPPLEMENTARY METHODS

Sample storage, DNA extraction, whole genome sequencing and variant calling

For the Pennsylvania transect, a small amount of blood (30-100ul) was drawn upon capture through brachial vein puncture using capillary tubes. Blood samples were either frozen or stored in Queen's lysis buffer until extraction. For the Missouri transect, the chickadees were euthanized and immediately frozen on dry ice, and later sampled for liver, heart, pectoralis tissue and skins for voucher specimens (all deposited at the University of Kansas Biodiversity Institute). Specimens were frozen on dry ice in the field and tissues were preserved in liquid nitrogen at the time of specimen preparation. DNA was extracted using Qiagen DNeasy blood and tissue kit following manufacturer's recommendations or using salt extraction following (Funk et al. 2021) and quantified using an Invitrogen Qubit 3.0 fluorometer.

Whole genome library preparation was performed using a Nextera XT DNA Library Preparation Kit following standard protocol. Libraries were pooled and sequenced on an Illumina NovaSEQ 6000 (paired-end reads, 150 base pairs) to approximately 15x coverage at the University of Colorado Cancer Center Genomics and Microarray Core Facility. We trimmed low quality reads and removed Illumina adapters using Trimmomatic 0.39 (Bogler et al. 2014) and performed quality control on trimmed sequence files using FastQC 0.11.7 (Andrews 2010). Next, bwa mem v.0.7.17-r1188 (Li 2013) was used to align reads to the chromosome-scale mountain chickadee reference genome (a genome from closely related species was used to avoid reference bias in genotype calls) and annotation (Semenov et al. 2024). Bam files were sorted, duplicates were marked, and files were indexed using samtools v. 1.3.1 (Li et al., 2009) and picard-tools v.2.8.1 (Broad Institute 2019). Variants were called using HaplotypeCaller from GATK v.4.2 (Poplin et al. 2007). The resulting gvcfs were merged using CombineGVCFs and genotyped with GenotypeGVCFs followed by VariantFiltration using GATK-recommended filters. We used VCFtools v.0.1.15 (Danecek 2011) to remove indels, keep only biallelic SNPs with minor allele frequency above 5%, minQ>20, min-meanDP>4, max-meanDP<75 and max-missing=0.75

Simulations of the hybrid zone dynamics

Previous findings of variation in the rate of chickadee hybrid zone movement (Alexander et al., 2022) suggested that there are greater opportunities for bidirectional introgression in slowly moving hybrid zones, which permit an increased number of generations of hybrids and backcrosses to go through the process of allelic segregation. Inspired by corroborating evidence from the present study (see Results), we tested the hypothesis that a faster moving hybrid zone can result in more restricted genome-wide introgression (i.e., steeper genomic clines), using forward-time spatial simulations implemented in SLiM v3.7.1; (Haller et al. 2019). We modeled a 200 base pair genome as four 50 bp chromosomes. Three of these chromosomes contained only neutral mutations, while the fourth contained a mutation under selection. We simulated 1000 diploid individuals (Figure S6) under a scenario of secondary contact in XY-coordinate space and imposed selection based on the X-coordinate of the individual. This allowed selection to differ between sides of a contact zone (set initially at X=0) and change over time as the contact

zone moved. The strength of selection was drawn randomly each iteration from a uniform distribution ranging from 0.01 to 0.45. The speed at which the contact zone moved was also randomly drawn from a uniform distribution ranging from 1/50 to 1/250, translating to the number of generations it takes for the contact zone to move 1 unit along the X-axis. Each simulation was run for 200 generations of burn-in to allow some diffusion of neutral mutations before the contact zone began to move. We ran 10,000 iterations of the simulation and output the results of each iteration as a *vcf* file by sampling individuals from 20 artificial populations divided by coordinate space along the X-axis. As with our empirical data, we used *gghybrid* (Bailey et al. 2023) to generate cline rates from simulation results, implementing the analysis with identical MCMC parameters. Due to the stochasticity of the spatial movements in the simulations, we removed iterations with an insufficient number of hybrids (~1%). We further tested for statistical correlations between the speed of contact zone movement and the steepness of the cline, while controlling for strength of selection using a linear model in R v.3.6.1.

Connection between climate and spatiotemporal dynamics of hybridization
To examine whether climatic differences between transects might contribute to the observed pattern of more restricted introgression in Pennsylvania than Missouri (see Results), we compared climate variables in R version 4.3.3 (R Core Team 2024) with PCA. We first downloaded the WorldClim 2.1 bioclimatic dataset (https://worldclim.org/data/worldclim21.html; Fick and Hijmans 2017) at 2.5-minute resolution. This dataset contains 19 bioclimatic variables derived from monthly averages from 1970-2000 at a resolution of 2.5 minutes (i.e., approximately 4.5 km^2 cells at the equator). Given previous knowledge about chickadee biology (e.g., Brittingham and Temple 1988, Desrochers et al. 1988, Benedict et al. 2020), and the results of our BGC analysis (see Results), our hypothesis was that *harsher winters are associated with stronger selection*; hence, stronger effects of climatic factors are expected in Pennsylvania compared to Missouri. We focused our analysis on six bioclimatic variables that describe precipitation levels, cold temperatures, and temperature fluctuations. We used the *extract()* function from the R package *raster* (version 3.6-26; Hijmans 2023) to extract mean diurnal range, isothermality, minimum temperature of the coldest month, mean temperature of the coldest quarter, annual precipitation, and precipitation of the coldest quarter data for cells in and around the approximate center of the Pennsylvania and Missouri hybrid zones. Specifically, we selected data for all cells within 50 km of Hawk Mountain, PA (-75.98706 degrees E, 40.63439 degrees N; *N* = 481 locations) and 50 km of Rockville, MO (-94.1213 degrees E, 38.0639 degrees N; *N* = 463 locations).

We first examined the Pearson correlation between our six focal climate variables using the R function *cor()*, Next, we used the R function *prcomp()* to perform PCA with scaled variance, a setting implemented to reduce the high dimensionality of climate data. Because some of our focal variables were highly correlated, we conducted a second PCA using only those variables that showed little correlation (see Supporting Information). We then examined differences between key climate variables that loaded heavily on PCI; because the distributions of these variables were non-normal (based on output from the R function *shapiro.test()*) and had unequal variances across groups

(based on output from the R function *bartlett.test()*), we tested for significant differences in overall transect climate using a nonparametric Wilcoxon rank sum test (*wilcox.test()*). Because some of our focal variables are highly correlated (Table S5), we conducted a second PCA that contained only relatively uncorrelated variables. Using the *prcomp* R function, we conducted a principal component analysis (PCA) with scaled variance to reduce the dimensionality of this dataset using three climate variables: *isothermality*, *mean temp coldest quarter*, and *precipitation of the coldest quarter*.

SUPPLEMENTARY RESULTS

Selection strength and hybrid zone movement both contribute to cline steepness

Forward-time simulations to reconstruct genomic clines in a dynamic hybrid zone with variable selection strength and rate of movement revealed significant interaction effects between model parameters. Variation in selection strength and the speed at which the contact zone moved resulted in variable steepness of the genomic cline (Figure S7). At low selection strengths, we observed a negative relationship between cline steepness and contact zone movement speed, indicating that faster moving contact zones produce steeper clines (Figure S7). In simulations with stronger selection, cline steepness was either stable or was positively correlated with contact zone movement speed. Importantly, there was high variance in the estimated cline steepness across similarly parameterized simulations. Median cline steepness across iterations was estimated at 5.7 with a standard deviation of 31. However, roughly 2% of clines had values greater than 65 (approx. 2 standard deviations), with one estimate as high as 600, producing a long tail on the distribution. Such extreme estimates of cline steepness are likely to be an artifact of the estimation step in *gghybrid*, potentially due to uncertainty or failure to converge, rather than a biologically meaningful result. To explore the influence of these potential outliers we therefore ran linear models using both the full set of estimated cline steepness, and a filtered set that removed estimates above 65. While data processing did not change the trend of the relationship between predictor variables and cline steepness, it did influence the significance of the relationship described above. Linear models using the filtered set found all three terms to be significant predictors of cline steepness (selection coefficient, contact zone movement speed, and their interaction), while only selection was found to be a significant predictor in the unfiltered set. In both linear models, the effect of contact zone movement speed was small (filtered slope=-0.009, p=0.043; unfiltered slope=-0.006, p=0.61; see Table S2 for full results).

Table S2: Simulation results

Linear model of simulation results					
		Unfiltered		Filtered*	
Model	Term	Coefficient	P	Coefficient	P
s+r+(s*r)	s	37.06	**3.78E-08**	28.6	**< 2e-16**
	r	-0.006	0.61151	-0.009	**0.041923**
	s*r	0.07	0.08823	0.05	**0.000633**
Unfiltered R^2=0.04					

Filtered R^2=0.14
r = rate of contact zone movement
s = selection coefficient
* Outlier cline rates (v>65) removed from analysis

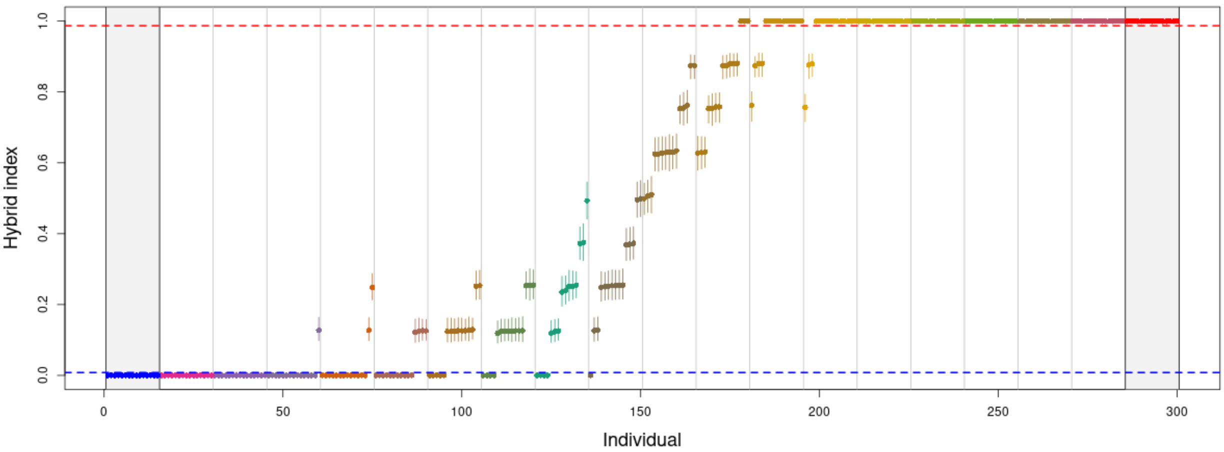

Figure S6. Hybrid index of simulated individuals demonstrating that admixture in our simulations produced a variety of genomic backgrounds spanning multiple hybrid classes, including F1s, backcrosses, and intermediate classes in both directions. We view these results as evidence that our simulation parameters and genome structure was sufficient for calculating genomic clines.

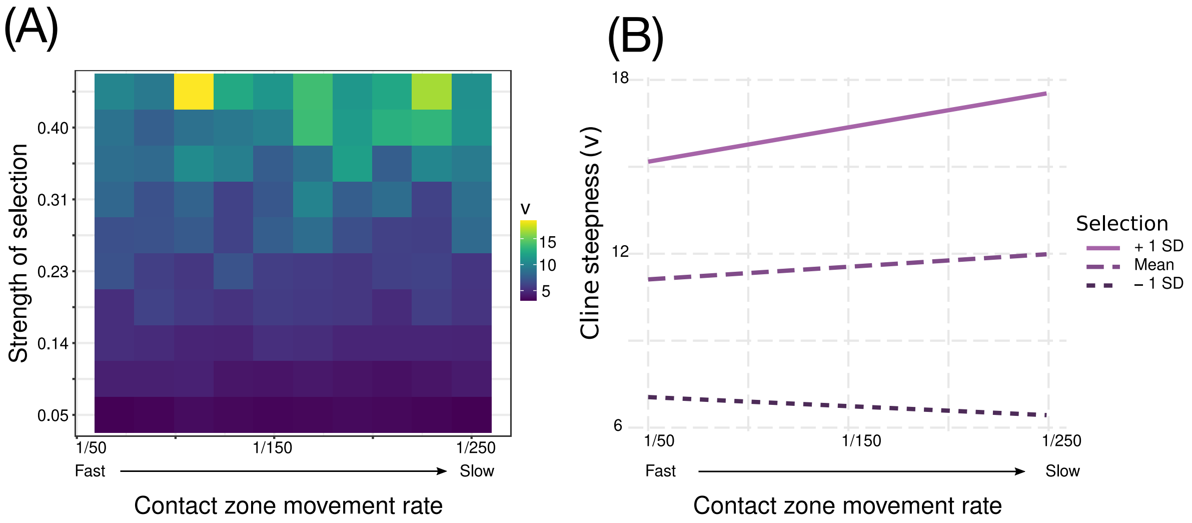

Figure S7. Simulations of the genomic pattern dynamics in a moving hybrid zone with variable strengths of selection and moving rates. (A) Heatmap summarizing 10,000 estimated cline rates across different combinations of selection strengths and contact zone movement rate. Movement rate is shown as distance moved per generation along the x-axis of simulation space. The *v* parameter is cline steepness, with higher values indicating steeper clines (more rapid transition in allele frequency compared to

genome-average). (B) Relationship between cline steepness and contact zone movement rate. Note that cline steepness and contact zone movement rate are reversely related and depend on selection strength.

Environmental variables differ between Pennsylvania and Missouri
Sites around the centers of the Pennsylvania and Missouri hybrid zones clustered separately in the environmental PCA (Figure S8). Mean temperature of the coldest quarter, precipitation of the coldest quarter, and mean diurnal range loaded most heavily on PC1, with isothermality loading most heavily on PC2 (Table S3). While sites from Pennsylvania and Missouri showed strong variation and overlapped on PC2, PC1 clearly differentiated the two transects: sites from Pennsylvania had negative PC1 values, while sites from Missouri had positive PC1 values (Figure S8). Precipitation variables loaded negatively on PC1, while temperature variables loaded positively on PC1 (Table S4); sites from Pennsylvania were colder ($P<0.001$; Figure S8), experienced more precipitation ($P<0.001$; Figure 6C), and showed smaller daily temperature fluctuations ($P<0.001$; Figure S8) than sites from Missouri between 1970 and 2000.

Sites around the centers of the Pennsylvania and Missouri hybrid zones clustered separately on the PCA (Figure S9). Mean temperature of the coldest quarter and precipitation of the coldest quarter loaded most heavily on PC1 and isothermality loaded most heavily on PC2 (Table S5). While sites from Pennsylvania and Missouri showed a great deal of variation and overlap on PC2, PC1 differentiated between sites along these transects (Figure S9). Precipitation variables loaded negatively on PC1, while temperature variables loaded positively on PC1 (Table S4); sites from Pennsylvania were colder ($P<0.001$; Figure S8), experienced more precipitation ($P<0.001$; Figure S8), and showed smaller daily temperature fluctuations ($P<0.001$; Figure S8) than sites from Missouri between 1970 and 2000 (Figures S8).

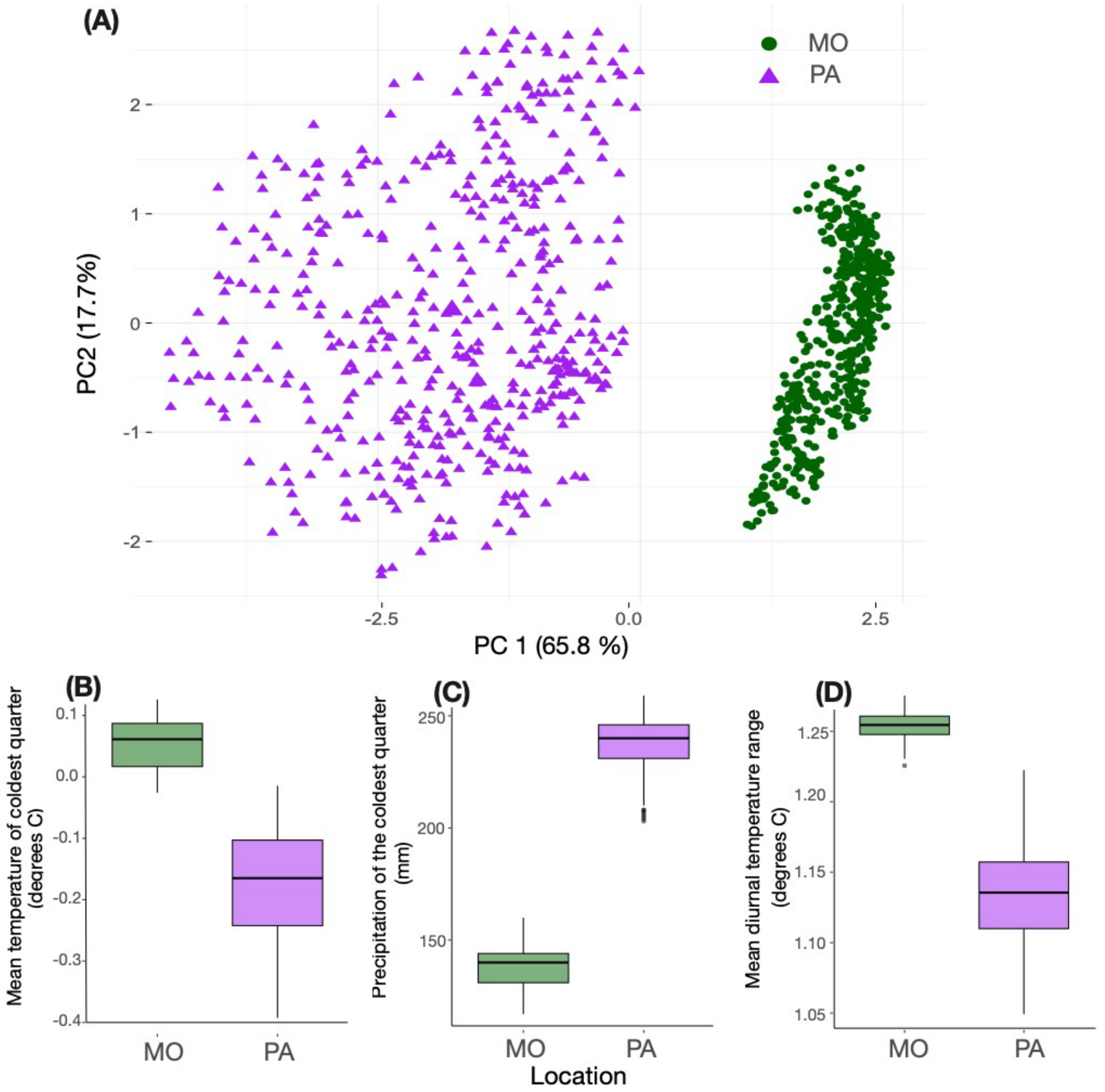

Figure S8. Climatic differences between the Missouri and Pennsylvania hybrid zones. (A) A principal component analysis of six climatic variables separates meteorological sites in and around the approximate center of the Pennsylvania hybrid zone (N = 481; purple triangles) from sites in and around the approximate center of the Missouri hybrid zone (N = 463; green circles) along the PC1 axis. Mean temperature of the coldest quarter (B), precipitation of the coldest quarter (C), and mean diurnal temperature range (D) load most heavily on PC1 and differ significantly between Pennsylvania and Missouri sites. Boxplots (B-D) show a median (center line), first quartile (bottom of box), third quartile (top of box), and minimum and maximum (whiskers). Outliers are represented as solid points.

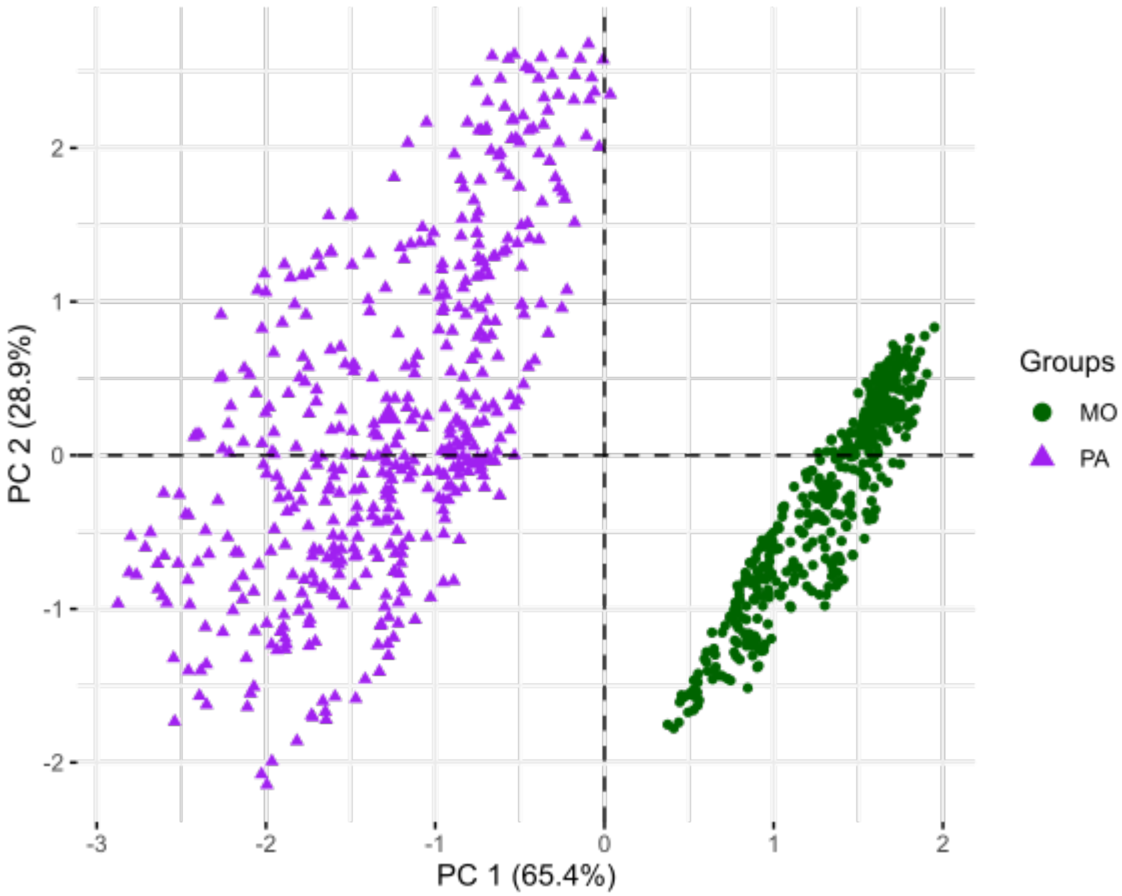

Figure S9. Climatic differences between Pennsylvania and Missouri: A principal component analysis of three climatic variables, isothermality, mean temperature of the coldest quarter (°C), and precipitation of the coldest quarter (mm), separates meteorological sites in and around the approximate center of the Pennsylvania hybrid zone (N = 481; purple triangles) from sites in and around the approximate center of the Missouri hybrid zone (N = 463; green circles). Mean temperature of the coldest quarter and precipitation of the coldest quarter load most heavily on PC1: sites with large, positive values are colder and drier, while sites with large, negative values are warmer and wetter. Isothermality loads most heavily on PC2.

Table S3: Variance percentage explained by each climate variable for each of the first four PC axes (explaining 99.7% of total variance). Variables with high loadings (>20%) are highlighted for PC 1 and PC 2.

	PC 1 (65.85%)	PC 2 (17.73%)	PC 3 (13.18%)	PC 4 (2.92%)
mean diurnal range (°C)	0.21	0.05	0.14	0.06
isothermality	0.05	0.75	0.002	0.1

minimum temperature of the coldest month (˚C)	0.13	0.0008	0.61	0.0007
mean temperature of the coldest quarter (˚C)	0.23	0.0006	0.08	0.07
annual precipitation (mm)	0.18	0.16	0.04	0.62
precipitation of the coldest quarter (mm)	0.21	0.04	0.12	0.15

Table S4: Variable loadings (eigenvectors) on the first four PC axes (explaining 99.7% of total variance).

	PC 1 (65.85%)	PC 2 (17.73%)	PC 3 (13.18%)	PC 4 (2.92%)
mean diurnal range (˚C)	0.46	0.22	0.37	-0.24
isothermality	0.21	0.87	0.05	0.32
minimum temperature of the coldest month (˚C)	0.36	-0.03	-0.78	0.03
mean temperature of the coldest quarter (˚C)	0.48	-0.03	-0.29	-0.26
annual precipitation (mm)	-0.42	0.40	-0.20	-0.79
precipitation of the coldest quarter (mm)	-0.46	0.21	-0.35	0.39

Table S5: Pearson correlation between six focal climate variables.

	mean diurnal range (˚C)	isothermality	minimum temperature of the coldest month (˚C)	mean temperature of the coldest quarter (˚C)	annual precipitation (mm)	precipitation of the coldest quarter (mm)
mean diurnal range (˚C)						
isothermality	0.58					
minimum temperature of the coldest month (˚C)	0.41	0.25				
mean temperature of the coldest quarter (˚C)	0.79	0.36	0.86			

annual precipitation (mm)	-0.69	-0.04	-0.49	-0.73		
precipitation of the coldest quarter (mm)	-0.89	-0.19	-0.43	-0.81	0.84	

Table S6: Variance percentage explained by each climate variable for all three PC axes.			
	PC 1 (65.42%)	PC 2 (28.86%)	PC 3 (5.72%)
isothermality	0.14	0.83	0.03
mean temperature of the coldest quarter (℃)	0.45	0.21	0.52
precipitation of the coldest quarter (mm)	0.41	0.14	0.45

Table S7: Variable loadings (eigenvectors) on PC axes.			
	PC 1 (65.42%)	PC 2 (28.86%)	PC 3 (5.72%)
isothermality	0.37	0.91	-0.16
mean temperature of the coldest quarter (℃)	0.67	-0.15	0.72
precipitation of the coldest quarter (mm)	-0.64	0.38	0.67

SUPPLEMENTARY REFERENCES

1. Alexander A, Robbins MB, Holmes J, Moyle RG, Peterson AT. Limited movement of an avian hybrid zone in relation to regional variation in magnitude of climate change. Mol Ecol. 2022 Dec;31(24):6634-6648. doi: 10.1111/mec.16727. Epub 2022 Oct 21. PMID: 36210655; PMCID: PMC9729445.

2. Andrews S. FastQC: a quality control tool for high throughput sequence data. Available online at: http://www.bioinformatics.babraham.ac.uk/projects/fastqc (2010).

3. Bailey, RI, Bayesian hybrid index and genomic cline estimation with the R package gghybrid (2023), Molecular Ecology Resources, 00, 1–15. https://doi.org/10.1111/1755-0998.13910.

4. Benedict L. M., A. M. Pitera, C. L. Branch, D. Y. Kozlovsky, B. R. Sonnenberg, E. S. Bridge & V. V. Pravosudov 2020. Elevation-related differences in annual survival of adult food-caching mountain chickadees are consistent with natural selection on spatial cognition. Behavioral Ecology and Sociobiology.

5. Bolger et al. Trimmomatic: A flexible trimmer for Illumina Sequence Data. Bioinformatics 30:2114-2120 (2014).

6. Brittingham MC, Temple SA. 1988. Impacts of Supplemental Feeding on Survival Rates of Black-Capped Chickadees. Ecology. https://doi.org/10.2307/1941007

7. Broad Institute, 'Picard Tools', Broad Institute, Accessed 05/01/2019. http://broadinstitute.github.io/picard. (2019).

8. Danecek P et al. The Variant Call Format and VCFtools. Bioinformatics 27:2156-2158 (2011).

9. Desrochers A, Susan J. Hannon, Kelly E. Nordin, Winter Survival and Territory Acquisition in a Northern Population of Black-Capped Chickadees, *The Auk*, Volume 105, Issue 4, October 1988, Pages 727–736, https://doi.org/10.1093/auk/105.4.727

10. Fick, S.E. and R.J. Hijmans (2017. WorldClim 2: new 1km spatial resolution climate surfaces for global land areas. International Journal of Climatology 37 (12): 4302-4315.

11. Funk, E.R., Mason, N.A., Pálsson, S., Albrecht, T., Johnson, J.A., Taylor, S.A. A supergene underlies linked variation in color and morphology in a Holarctic songbird. Nat Commun. 2021 Nov 25;12(1):6833. doi: 10.1038/s41467-021-27173-z. PMID: 34824228; PMCID: PMC8616904.

12. Haller, B.C., and Messer, P.W. (2019). SLiM 3: Forward genetic simulations beyond the Wright–Fisher model. Molecular Biology and Evolution 36(3), 632–637. DOI: https://doi.org/10.1093/molbev/ msy228

13. Hijmans R (2023). _raster: Geographic Data Analysis and Modeling_. R package version 3.6-26, <https://CRAN.R-project.org/package=raster>.

14. Li H. Aligning sequence reads, clone sequences and assembly contigs with BWA-MEM. arXiv:1303.3997v1 (2013).

15. Li et al. The Sequence Alignment/Map Format and SAMtools. Bioinformatics 25:2078-2079 (2009).

16. Poplin et al. Detailed description of HaplotypeCaller; best reference for germline joint calling. bioRxiv (2017).
